# Nanoplasmonic Single‐Tumoroid Microarray for Real‐Time Secretion Analysis

**DOI:** 10.1002/advs.202401539

**Published:** 2024-06-24

**Authors:** Yen‐Cheng Liu, Saeid Ansaryan, Jiayi Tan, Nicolas Broguiere, Luis Francisco Lorenzo‐Martín, Krisztian Homicsko, George Coukos, Matthias P. Lütolf, Hatice Altug

**Affiliations:** ^1^ Bionanophotonic Systems Laboratory Institute of Bioengineering School of Engineering École Polytechnique Fédérale de Lausanne Lausanne 1015 Switzerland; ^2^ Laboratory of Stem Cell Bioengineering Institute of Bioengineering School of Life Sciences and School of Engineering École Polytechnique Fédérale de Lausanne Lausanne 1015 Switzerland; ^3^ Department of Oncology Centre Hospitalier Universitaire Vaudois Rue du Bugnon 46 Lausanne 1005 Switzerland; ^4^ Ludwig Institute for Cancer Research Ludwig Lausanne Branch Chem. des Boveresses 155 Epalinges 1066 Switzerland; ^5^ Swiss Cancer Center Leman Rue du Bugnon 25A Lausanne 1011 Switzerland; ^6^ Agora Translational Research Center Rue du Bugnon 25A Lausanne 1011 Switzerland

**Keywords:** microarray, microfluidics, nanophotonics, nanohole array, nanoplasmonic biosensor, protein secretion, tumoroids

## Abstract

Organoid tumor models have emerged as a powerful tool in the fields of biology and medicine as such 3D structures grown from tumor cells recapitulate better tumor characteristics, making these tumoroids unique for personalized cancer research. Assessment of their functional behavior, particularly protein secretion, is of significant importance to provide comprehensive insights. Here, a label‐free spectroscopic imaging platform is presented with advanced integrated optofluidic nanoplasmonic biosensor that enables real‐time secretion analysis from single tumoroids. A novel two‐layer microwell design isolates tumoroids, preventing signal interference, and the microarray configuration allows concurrent analysis of multiple tumoroids. The dual imaging capability combining time‐lapse plasmonic spectroscopy and bright‐field microscopy facilitates simultaneous observation of secretion dynamics, motility, and morphology. The integrated biosensor is demonstrated with colorectal tumoroids derived from both cell lines and patient samples to investigate their vascular endothelial growth factor A (VEGF‐A) secretion, growth, and movement under various conditions, including normoxia, hypoxia, and drug treatment. This platform, by offering a label‐free approach with nanophotonics to monitor tumoroids, can pave the way for new applications in fundamental biological studies, drug screening, and the development of therapies.

## Graphical abstract

Real‐time secretion analysis from single tumoroids is enabled by a label‐free spectroscopic imaging platform with an advanced optofluidic nanoplasmonic biosensor. Demonstrated with colorectal tumoroids to investigate their vascular endothelial growth factor secretion, growth, and movement under various conditions, this platform shows its capability of monitoring secretion dynamics, motility, and morphology and promises new avenues for potential screening applications.

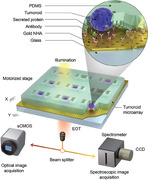

## 1 Introduction

Organoid research has gained significant importance since its debut a few decades ago, and recent advancements have greatly accelerated its development. In particular, tumor organoids (tumoroids) have gathered substantial attention in cancer research due to their 3D architecture.^[^ ^1^, ^2^, ^3^ ^]^ In comparison to 2D cell culture, tumoroids closely resemble the natural cell organization within a tumor, thereby providing a more representative in vitro model.^[^ ^4^ ^]^ Notably, the oxygen and nutrient gradients present in tumoroids more closely resemble those observed in actual tumors, thus better mimicking cell proliferation, metabolism, and response to therapies.^[^ ^5^, ^6^ ^]^ As a result, tumoroids hold great promise in advancing cancer research and facilitating personalized medicine strategies including cell‐based therapies,^[^ ^7^ ^]^ immunoengineering,^[^ ^8^, ^9^ ^]^ and nanomedicines.^[^ ^10^ ^]^

To date, there exist various approaches to form organoids, including hanging drop,^[^ ^11^ ^]^ scaffold‐based culture,^[^ ^12^, ^13^ ^]^ spinning flask,^[^ ^14^ ^]^ microfluidics,^[^ ^15^, ^16^ ^]^ and wettability patterning approach.^[^ ^17^, ^18^ ^]^ Among these, microfluidics stands out as a well‐established technology widely utilized for in situ studies of living organisms.^[^ ^19^, ^20^, ^21^ ^]^ With the advantages of increased precision, easy automation, and parallelization, microfluidics technology particularly accelerates the development of tumoroid studies, not only in terms of formation but also in situ analysis due to the ease of integration with biosensors.^[^ ^22^ ^]^ The precise fluidic manipulation also enables the interaction studies between the organoids and different types of cells, for instance, immune cells.^[^ ^23^ ^]^ More importantly, microfluidic devices can be used to deliver drugs and other compounds to specific regions of the organoid, allowing researchers to study how they respond to different treatments.^[^ ^16^, ^24^ ^]^

The organoids can be analyzed with various imaging approaches to evaluate their behavior and response to drugs. Since morphological analysis is foundational in organoid evaluation, techniques like bright field microscopy revealing architectural details and cell organization can shed light on how cells interact and differentiate within the 3D environment.^[^ ^25^, ^26^ ^]^ Organoid viability can be also assessed with fluorescence microscopy and dyes visualizing live and dead cells, thus offering insights into the health and effects of experimental conditions or drugs. Combining this with time‐lapse microscopy can enable dynamic tracking of cell viability over time and capture responses to changing factors.^[^ ^27^, ^28^ ^]^ Beyond visual observation, genomic analysis can be conducted to profile gene expression in tumoroids subjected to different conditions or drug treatments, revealing signaling pathways and regulatory networks governing organoid behavior.^[^ ^29^, ^30^, ^31^ ^]^

In addition to the aforementioned approaches, monitoring the secretion from organoids is of significant importance for their functional analysis either for disease modeling, fundamental studies of organ physiology, or drug screening as secretion reflects the presence of specific proteins, genes, or metabolites. This information can be used to better understand the biology of the organoids, and to identify potential targets for therapeutic intervention. The analysis of secreted proteins in the extracellular environment can be performed with immunoassay techniques such as ELISA by sampling conditioned medium from the culture of an organoid population at selected time points.^[^ ^32^, ^33^ ^]^ However, the end‐point measurement nature of immunoassays imposes limitations on capturing real‐time secretion dynamics. Fluorescence imaging can be an alternative to study single organoids. A recent work utilizes fluorescence microscopy with microfluidic droplet technology to measure protein secretion,^[^ ^34^ ^]^ although real‐time monitoring remains an unmet challenge in this context. In addition, fluorescence techniques require laborious multi‐step labeling protocols and reagents. Therefore, there remains a clear need for new platforms that can enable functional analysis by measuring the dynamics of secreted proteins and visualization of single living organoids in real‐time and in a high‐throughput manner to gain comprehensive insights into their phenotypic behavior.

Here, we present a label‐free microarray for real‐time detection of protein secretion from individual tumoroids using a nanoplasmonic biosensor integrated with a customized polymeric microwell. The optical setup employs a synchronized dual‐imaging scanning platform that allows simultaneous collection of spectroscopic and bright‐field images from multiple positions to investigate tens of tumoroids in a microarray format. The unit cell of the open‐top polymeric microwell contains two detection chambers on both sides of the organoid well, connected by a channel featuring micropillar arrays. This novel design allows the passage of secreted proteins while effectively blocking escaping cells or debris from reaching the detection chamber, ensuring accurate plasmonic signal acquisition.

We validate our sensing platform by monitoring the secretion of vascular endothelial growth factor A (VEGF‐A), a protein critical in angiogenesis and tumor growth through the formation of new blood vessels,^[^ ^35^, ^36^ ^]^ from colorectal cancer (CRC) cell line‐derived tumoroids. Furthermore, we can analyze VEGF‐A secretion from clinically relevant CRC patient‐derived tumoroids (PDT). The binding of secreted VEGF‐A on the sensor surface is sensitively detected from the acquired time‐resolved spectroscopic images by tracking the wavelength changes in the extraordinary optical transmission (EOT) spectrum of the gold nanohole array (AuNHA)‐based plasmonic biosensor to capture secretion kinetics over 20 h with 10 min time interval. Our results reveal significant variations in secretion levels and secretion rates among individual tumoroids under different conditions, including normoxia, hypoxia, and treatment with actinomycin D (ActD), an antibiotic known for its transcription inhibition and anticancer properties.^[^ ^37^ ^]^ By employing machine learning‐assisted processing of time‐resolved bright‐field tumoroid images, we achieve automated tumoroid recognition to study dynamic changes in size and motility. Notably, from this concurrent morphology analysis we observe a delay in tumoroid growth or a reduction in size following ActD treatment, supporting the anticancer efficacy of this drug. The suppressed tumoroid growth associated with drug treatment correlates with lower VEGF‐A secretion under the same conditions.

The developed microarray platform enables real‐time measurement of secreted proteins from multiple individual tumoroids in parallel at high temporal resolution and over a long observation period. Integrating nanophotonics and microarray‐based microfluidics, this technology has the advantage of facilitating a comprehensive study of organoid secretion, morphology, and motility. It holds immense potential to investigate in real‐time the intricate dynamic relationship between tumoroid behavior and protein secretion in a high‐throughput manner and can serve as a valuable tool for various applications such as fundamental tumoroid research and the development of novel anticancer treatments.

## 2 Results and Discussion

### 2.1 Optical Setup for Single‐Tumoroid Secretion and Morphology Analysis

**Figure 1:**
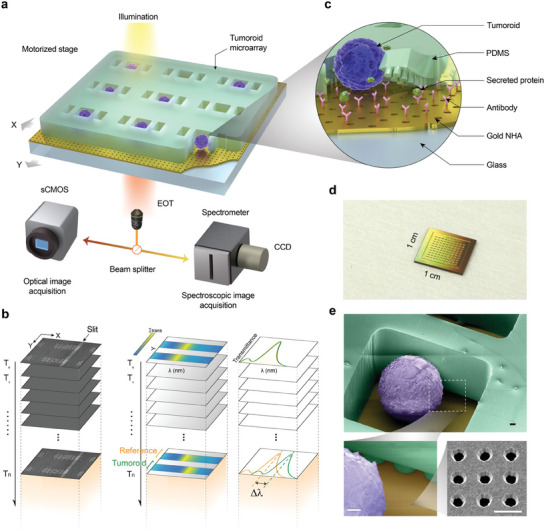
Optical setup and detection principle for single‐tumoroid secretion analysis. a) Schematic illustration of the dual‐imaging spectroscopic scanning system with an integrated optofluidic biosensing chip. The transmitted light collected by the objective lens enters a beam splitter and simultaneously goes to a spectrometer for spectral analysis of the gold nanohole array transmission response to detect the secretion signal and a sCMOS camera for optical image collection for observing and analyzing tumoroid morphology. The motorized stage is controlled by a user interface to repeatedly scan over pre‐defined positions, enabling the measurement of multiple individual tumoroids in a microarray format. b) An illustrative principle of simultaneous spectroscopic and optical tumoroid monitoring. The system collects images at pre‐defined positions repeatedly, forming image stacks. The optical image stack is used to analyze tumoroid morphology, size, and centroid, while the spectroscopic image stack is used to analyze the target protein secretion by monitoring in real‐time the peak wavelength shift (Δ*λ*) in the resonant optical transmission response of the NHA resulting from the binding of protein on the biofunctionalized sensor surface. c) A close‐up view of the microarray unit highlights the PDMS parts with tumoroid well and the micropillar‐array based channel connection to the two detection wells, seeded tumoroid, antibody functionalized plasmonic nanohole array chip, and the target secreted protein. d) The photo of an assembled biochip with the PDMS microwell array attached to the AuNHA substrate. e) A colored scanning electron microscope (SEM) image of the CRC tumoroid on the AuNHA chip in a microwell (top panel, scale bar = 10 µm), and a zoomed‐in SEM image showing both the micropillars in the channel and the nanoholes (lower left panel, scale bar = 10 µm) with a close‐up view of the nanoholes (lower right panel, scale bar = 600 nm).

The real‐time and label‐free detection of single‐tumoroid secretion is achieved using an optical setup similar to that described previously.^[^ ^38^ ^]^ As shown in the schematic illustration in **Figure** **1**a, the nanoplasmonic chip on the microscope stage is illuminated by light from a broadband tungsten‐halogen lamp, filtered by a band‐pass filter (with a central wavelength of 860 nm and a FWHM of 120 nm). A 10× objective lens collects the transmitted signal, which is then split by a dual‐camera image splitter into two directions. The first beam is directed to a spectrometer connected with a deep‐cooled CCD camera to generate 1D spectroscopic images through a slit opening with a width of 500 µm and a grating with 600 lines mm^−1^. The second beam is directed to a monochrome CMOS camera to capture optical wide‐field images. Custom MATLAB graphical user interface is utilized for data acquisition, real‐time analysis, and display.

Spectroscopic and optical images of the transmitted light are collected at specified time intervals, which are set to 10 min for our measurements. The user interface controls the synchronization of the automated stage scanning over pre‐defined microwell positions repeatedly, creating image stacks of both spectroscopic and optical data at each microwell position for real‐time analysis and post‐processing. To monitor target protein secretion, a label‐free refractometric biosensing principle based on the EOT phenomenon occurring on the AuNHA is used with the acquired spectroscopic image stacks (Figure 1b). When secreted proteins bind specifically to antibodies pre‐functionalized on the sensor surface, the nearby refractive index increases, causing a redshift in the EOT resonance (Δ*λ*) in microwells with secreting tumoroids (Figure 1b, right panel). In contrast, empty microwells without tumoroids exhibit no such resonance shift, serving as references to correct for any systematic signal drift. Continuous tracking of spectral shifts from time‐resolved spectral image stacks allows us to generate a sensorgram for measuring secretion dynamics from multiple individual tumoroids. To monitor tumoroid morphology such as growth and the movement/mobility, the bright‐field optical image stacks are acquired and processed with segmentation techniques.

The integrated biosensor chip is composed of a fused silica substrate containing an optically thick gold film (≈120 nm) with nanohole array structures, and an open‐top microwell membrane made of Polydimethylsiloxane (PDMS) for isolating the tumoroids (Figure 1c). The nanoplasmonic structure is based on circular apertures in square lattice (200 nm diameter, and 600 nm periodicity). The chip is compact, with dimensions of 1 cm × 1 cm, enabling the measurement of at least 10 × 10 microwells with a 540 µm × 200 µm unit‐cell size (Figure 1d), each containing a single‐tumoroid (Figure 1e).

### 2.2 Microcompartment for Isolation of Tumoroids and Chip Configuration

**Figure 2:**
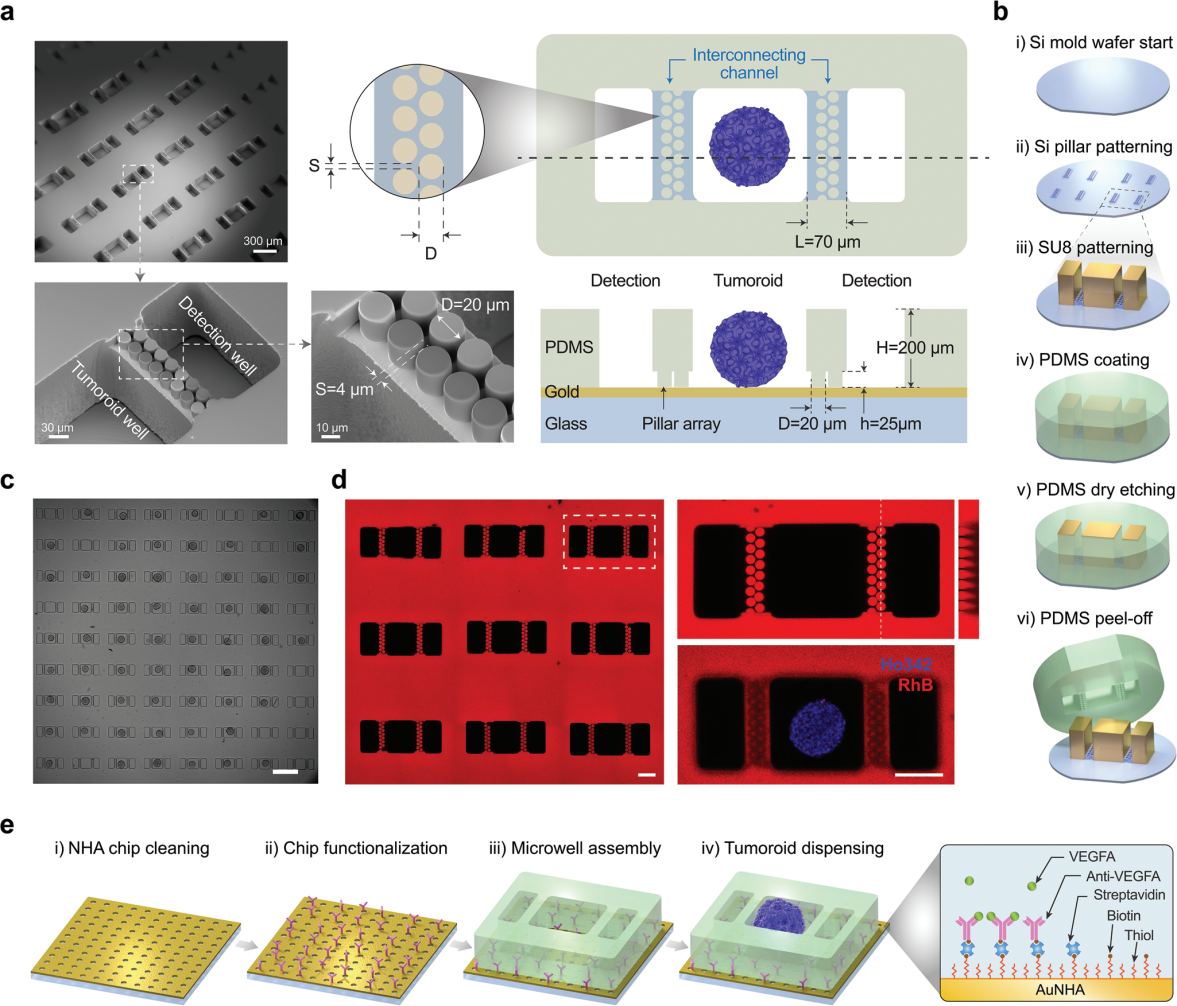
Design and fabrication of the open‐top polymeric microwell array, and chip configuration for tumoroid secretion analysis. a) An SEM image of a flipped PDMS microwell array of microwells (top left) and a zoomed‐in view of a single microwell unit showing the tumoroid well, detection well, and the connecting channel with the micropillar array in between (bottom left). The close‐up view of the micropillar array shows the optimized pillar size and spacing (bottom middle). The top view (top right panel) and cross‐sectional view (bottom right panel) of a single microwell unit composed of two detection wells and on tumoroid well provide a clearer illustration of the design. Close‐up view (top middle panel) shows the micropillar design parameters to block detaching cells or debris. S: spacing between pillars; D: diameter of pillars. Bottom panel: cross‐sectional view of the microwell. h: height of the micropillar; H: height of the tumoroid well and the detection well; L: space between the tumoroid well and the detection well. b) Schematics of the fabrication process for the PDMS microarrays. c) A microwell array seeded with HCT116 tumoroids. Scale bar = 500 µm. d) Fluorescence visualization of the microwells for tumoroid analysis: confocal microscope images of the PDMS microwell array soaked in Rhodamine B dye (providing red color) shows 3 × 3 wells (left panel), zoom‐in view of one microwell highlighted in a white dashed box with a YZ cross‐sectional view reconstructed by the confocal microscope (top right), and an image of a HCT116 tumoroid stained with Hoechst 33342 (providing blue color) seeded into the tumoroid well of the microarray unit (bottom right). e) Main steps of the experimental procedure for the measurement (i to iv) and the schematics of AuNHA surface functionalization for specific VEGF‐A detection (right panel).

To exclusively monitor a sensing area for the binding of the target analyte and ensure no signal disruption from the escaping cells and debris entry into the light path toward the spectrometer slit, a specially‐designed micro‐compartment with a two‐layer structure has been developed. Each unit well comprises a tumoroid well for trapping and observing the tumoroid, along with two smaller detection wells on both sides for monitoring the resonance shift of the nanohole array for secretion sensing. Between the tumoroid wells and the detection wells, there are interconnecting channels consisting of micropillar array (**Figure** **2**a). The pillar height (h = 25 µm), the pillar diameter (D = 20 µm), and the spacing (S = 4 µm) between the micropillars are designed and optimized to effectively block escaping cells and large debris while permitting the passage and diffusion of protein molecules to the detection wells (Figure S1, Supporting Information). The height of the entire microwell structure (H = 200 +/− 20 µm) for both the tumoroid well and the detection well is designed to exceed the height of the tumoroids.

The size of the tumoroid wells is a crucial factor, as they must be large enough to accommodate the tumoroids while small enough to provide sufficient analyte accumulation and sensitivity in the detection well. Our setup separates the tumoroid wells from the detection area by the distance of the interconnecting channel length (L = 70 µm). Since the entire chip is functionalized with antibodies, the analyte binding signal is more pronounced when the tumoroids are situated closer to the detection well. Therefore, it is ideal for the tumoroids to be positioned nearer the border next to the detection wells. This concept has been validated through an experiment that compared the secretion signals from similarly‐sized tumoroids in larger and smaller tumoroid wells (Figure S2, Supporting Information). Due to the variability in tumoroid sizes and locations within the microwells, the diffusion distance of the secreted target proteins entering the detection well and the corresponding signal is inevitably affected. To address this concern, we increased the number of detection wells, placing them on both sides of the tumoroid wells, and averaged their signal to create a representative signal curve for each tumoroid. This averaging process makes the tumoroid's specific location less crucial in the final signal (Figure S3, Supporting Information).

The two‐layer PDMS microwell mesh is fabricated with a replica molding technique using a master mold with two layers of structures realized by silicon etching and SU8 lithography, respectively. Afterward, the PDMS coating is applied, and dry etching is performed to open the top of the wells before the structure is peeled off and attached to the functionalized AuNHA surface for tumoroid secretion analysis (Figure 2b). Due to the distinct dimensions of the tumoroid wells and the detection wells, selective filling of the tumoroids only into the tumoroid wells is possible through simple manual seeding because the larger‐sized tumoroids cannot fit into the smaller‐sized detection wells (Figure 2c). To provide better visualization of the two‐layer microwell and the arrangement of the micropillars, the PDMS micromesh membrane is fluorescently stained by soaking it in Rhodamine B dye (red fluorescence) and imaged using a confocal microscope (Figure 2d).

Before the biochip is loaded with the tumoroids and ready for measurement, the surface is functionalized with antibodies to ensure that our nanohole array biosensor is capable of detecting the secreted proteins without non‐specific binding of irrelevant molecules onto the sensor surface. This process involves several steps, including gold thiolation, streptavidin binding, biotinylated antibody binding, and blocking (Figure 2e, rightmost panel). An in‐flow measurement shows a distinct signal increase in each functionalization step (Figure S4, Supporting Information), and a specificity test further validates the binding specificity of the sensor surface to VEGF‐A (Figure S5, Supporting Information). To assess the sensitivity and limit of detection of our biosensor, we performed a calibration by introducing recombinant human VEGF‐A proteins at various concentrations as standards in microfluidic channels integrated into the AuNHA chip (Figure S6, Supporting Information). Our results demonstrate that our biosensor exhibits outstanding sensitivity, with a limit of detection of ≈157 pg mL^−1^ for VEGF‐A detection. The total secretion amount from the tumoroids can theoretically correlate with spectral shift data using calibration curves. In our set‐up, although the acquired shift signal is linked to the total amount, it remains partial due to the limited monitoring area within each well by the slit. The dispensing of tumoroids into the microwell arrays takes place after the chip has been functionalized and the PDMS microwell membrane has been attached to it. Once immersed in the medium, the assembled chip is prepared for tumoroid seeding and subsequent measurements. (Figure 2e).

### 2.3 Formation and Characterization of Cell Line‐Derived and Patient‐Derived Tumoroids

The tumoroids used in this study are CRC tumoroids grown from both cell lines and patient‐derived tumor cells. The HCT116 human colorectal carcinoma cell line is used to form the “HCT116 tumoroids”. Patient‐derived human CRC cells are obtained from resections of human colorectal tissues to form the “patient‐derived tumoroids” (PDTs), with the three patients named MS, NW, and NS. The PDTs are typically grown and expanded using a standard Matrigel dome approach in growth factor‐reduced Matrigel. However, since this approach lacks control in terms of size variation, it is not suitable to provide standardized samples for microarray‐based setups.

**Figure 3:**
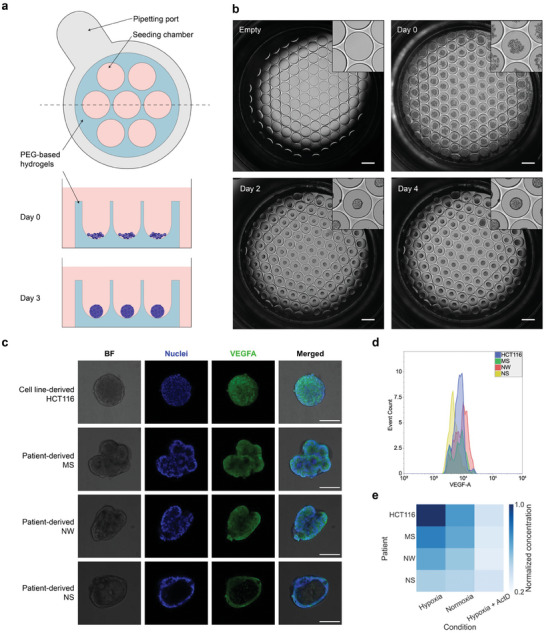
Formation of CRC tumoroids and characterization of VEGF‐A production. a) Schematic illustration of uniformly sized tumoroid formation using Grid3D plates. Top panel shows the illustrative top view of on well containing a microwell part and a pipetting port. Bottom panel shows the cross‐sectional view of cells seeded into the microwells in the beginning and after a few days when the cells aggregate and grow into a tumoroid. b) The bright‐field microscope images display a single well with 121 hydrogel microwells at various time points following cell seeding. The inset provides a zoomed‐in view of a selected microwell, presenting a clearer image of the formed tumoroid. Scale bar = 500 µm. c) Confocal fluorescence images of various types of tumoroids. Nuclei are stained with Hoechst 33342, and VEGF‐A is stained with secondary antibodies conjugated with DyLight‐488. Scale bar = 100 µm. d) Histogram illustrating the fluorescence signal of VEGF‐A from different tumoroid types under normoxic conditions from large‐particle flow cytometry analysis of the tumoroids. e) Heat map of VEGF‐A secretion from different patients under various conditions with Luminex assay.

To prepare uniform‐sized tumoroids, we used Grid3D technology, which features a 96‐well plate with hydrogel microcavity arrays at the bottom.^[^ ^12^ ^]^ This microcavity array is fabricated with poly(ethylene glycol) (PEG)‐based hydrogels, featuring U‐shaped bottoms that allow cells to aggregate during sedimentation (**Figure** **3**a). HCT116 cells and patient‐derived cells are dissociated from the culture flask and the Matrigel, respectively, and then diluted to an appropriate density before being manually seeded onto the hydrogel well array and cultured in a cell incubator. The patterned microwells with the same size ensure a similar number of cells landing into them. This ultimately leads to the formation of uniformly sized tumoroids under a suitable medium and growth duration (Figure 3b). The tumoroids form and grow larger with an extended culture duration and are collected for on‐chip measurements when their majority reaches a diameter of ≈150–200 µm. Bright‐field images of HCT116 tumoroids and the three PDTs in culture, imaged daily after seeding, are shown in Figure S7 (Supporting Information) to show the process of their growth.

From the generated tumoroids, we monitor the expression of VEGF‐A to validate our sensing platform. VEGF holds significance in cancer progression due to its role in initiating angiogenesis, the process of forming new blood vessels. This process is essential for tumors to access the vital nutrients and oxygen necessary for their development and potential metastasis.^[^ ^35^, ^39^ ^]^ VEGF‐A is in particular the predominant mediator of angiogenesis in the VEGF family.^[^ ^40^, ^41^ ^]^ To observe the morphology and assess the intracellular expression of VEGF‐A within them, tumoroids grown in the hydrogel microcavity arrays under normoxic condition are fluorescently labeled to stain VEGF‐A and the cell nuclei using an on‐array immunofluorescence staining procedure, followed by the imaging with a confocal microscope (Figure 3c). Comparing the HCT116 tumoroids and PDTs, we observe that the nuclei staining (blue) indicates a solid core full of cells in the HCT116 tumoroids, while lumens of various sizes are only visible in the PDTs. The observed lumen sizes between different patients are in accordance with the bright‐field images, with NS forming the largest cavities and MS forming the smallest ones. The VEGF‐A staining (green) shows intracellular VEGF‐A in both HCT116 tumoroids and PDTs, with the fluorescence signal located close to the cells. Although the fluorescence intensity may reflect the local density of the stained proteins,^[^ ^42^ ^]^ it is not common to quantify the intracellular protein directly based on the fluorescence signal due to multiple factors that might affect the signal intensity, such as cell density, cell distribution, and tumoroid size.

The fixed and stained tumoroids are analyzed with flow cytometry at the tumoroid level using a large particle flow cytometer to facilitate the comparison of intracellular VEGF‐A levels between different patient samples. A histogram illustrating event counts versus VEGF‐A intensity (Figure 3d) shows a lower VEGF‐A signal in NS PDT. This could be attributed to the cell density within it, resulting in reduced mean fluorescence intensity, provided that the intracellular expression in a single cell is not significantly different from that of other tumoroids. A graph with each patient sample in separate histograms is included in Figure S8 (Supporting Information).

To establish the capability of our platform for detecting extracellularly secreted VEGF‐A in response to various treatment conditions such as stimulation and inhibition, we perform the Luminex assay using a ProcartaPlex multiplexed plate and analyze the correlation of VEGF‐A levels in the supernatants of a population of HCT116 tumoroids and all the PDTs under the same conditions. The samples are prepared by culturing tumoroids on hydrogel microcavity arrays in a controlled manner.

To stimulate CRC tumoroids for VEGF‐A secretion, a hypoxic environment with 1% oxygen is implemented. When tumors experience hypoxia, the hypoxia‐inducible factor (HIF) pathway is activated, and the transcription of VEGF‐A is promoted by HIF to stimulate the formation of new blood vessels.^[^ ^35^, ^36^ ^]^ In addition to studying the stimulation of VEGF‐A secretion, an inhibition condition using Actinomycin D (ActD) is examined to reduce the secreted VEGF‐A amount. ActD is an FDA‐approved anticancer drug that directly binds to DNA and inhibits the expression of various genes, including VEGF‐A, by preventing the transcription of the VEGF‐A gene into messenger RNA (mRNA).^[^ ^43^, ^44^ ^]^ Previous studies have assessed the effect of ActD on VEGF‐A inhibition, tumor growth suppression, and cancer cell viability.^[^ ^45^, ^46^ ^]^ In our study, we use ActD with a concentration of 1 µg mL^−1^ to treat the tumoroids for one hour before replacing the drug with tumoroid expansion medium. The outcomes of the Luminex assay are illustrated in Figure 3e. As demonstrated, the hypoxic condition triggered a higher level of VEGF‐A secretion in comparison to the standard normoxic condition. On the other hand, the use of ActD at the given concentration and treatment duration led to a notably diminished VEGF‐A concentration.

### 2.4 VEGF Secretion Analysis from Single CRC Tumoroids on the Plasmonic Microarray

**Figure 4:**
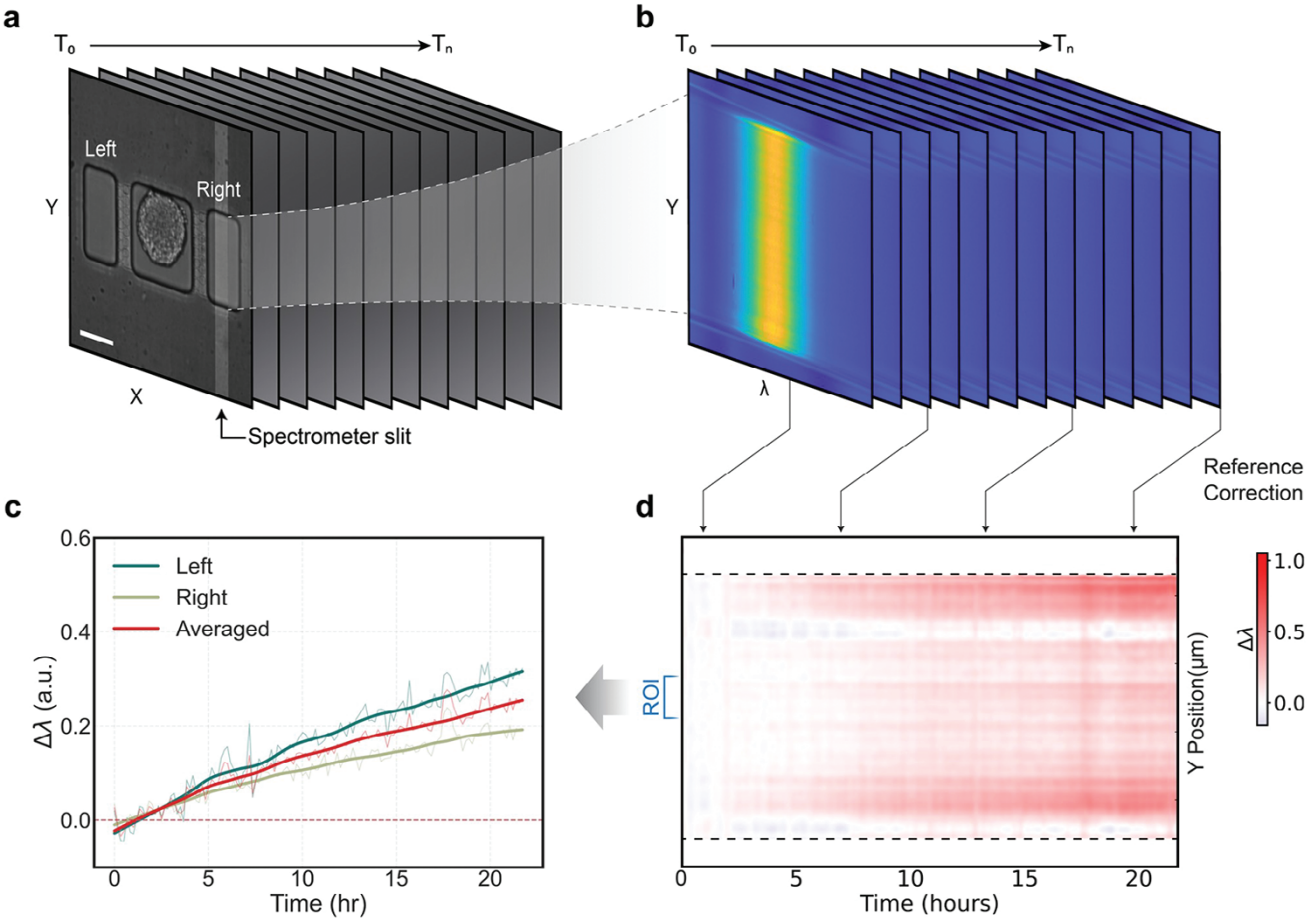
Spectroscopic image processing for spatiotemporal sensorgram construction. a) Illustration of an image stack showing the spectrometer slit opening highlighted. The collected light through the slit (aligned along Y axis) at different time points is dispersed by the spectrometer to provide spectral information and the corresponding time‐resolved spectroscopic image stack. Scale bar = 100 µm. b) The spectroscopic image stack is comprised of multiple frames over time, each capturing the spectral information along the slit direction and displaying it in X axis. c) Temporal sensorgram of the selected ROI from the detection well on the left and on the right as annotated in the spatiotemporal sensorgrams in panel d), and the averaged curve of the ROIs from left and right detection wells. Fainted lines (in c): raw data, solid lines (in c): smoothed data. d) The measured resonance peak centroid shift in pixels along the slit direction within the detection well is plotted in the Y axis to form the spatiotemporal sensorgram, where the value in each frame over time is depicted as color change along the X axis.

To monitor VEGF‐A secretion over time from individual tumoroids, we acquire time‐lapse spectroscopic images and analyze the spectra in each pixel along the spectrometer slit opening to measure the corresponding resonance peak centroid (**Figure** **4**a,b). By tracking the centroids and offsetting the data by the values at the initial time point of the measurement (T = 0), we obtain the spatiotemporal sensorgram in the detection well (Figure 4d). The horizontal axis shows time, the vertical axis (Y) shows position along the slit, and the color represents the centroid shift with respect to the value at T = 0. This shift reflects the amount of VEGF‐A binding on the functionalized sensor surface. The spatiotemporal sensorgram has a spatial resolution in the Y axis of ≈1.2 µm and the image acquisition time interval is set as 10 min. The data is subsequently upsampled and smoothed prior to plotting.

The monitoring of VEGF‐A secretion for a representative HCT116 tumoroid under normoxia condition over a period of 20 h is demonstrated, and the spatiotemporal sensorgrams are generated from both detection wells (Figure 4d). From each spatiotemporal sensorgram, a 20‐pixel (≈24.3 µm) region‐of‐interest (ROI) along the Y direction (slit direction) is selected to average the spectral shift value, generating the temporal sensorgram (Figure 4c). The thinner lines in the temporal sensorgram represent the calculated raw data, while the thick lines are smoothed curves. The flexibility of ROI position assignment helps to exclude pixel imperfections and signal disturbance from small debris that can sometimes appear in the detection well. The curves in the temporal sensorgram from the two detection wells are averaged to obtain the final curve (Figure 4c, red curve).

### 2.5 Profiling VEGF‐A Secretion from Single CRC Tumoroids upon Treatments

**Figure 5:**
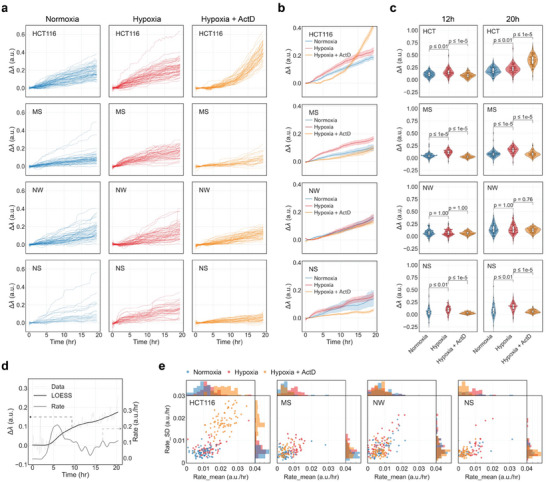
Kinetics of VEGF‐A secretion from CRC tumoroids at various conditions. a) Signal curves of VEGF‐A secretion from individual HCT116 tumoroids and all the PDTs (shown in different rows) under three different treatment conditions (shown in different columns), including normoxia, hypoxia, and hypoxia with the drug (ActD) treatment. b) An averaged curve along with the 95% confidence level plotted in the same graph for different types of tumoroids. c) VEGF‐A secretion level at specific time points for tumoroids under various conditions. Signal levels for each tumoroid are extracted at 12 h (left panels) and 20 h (right panels), and a statistical comparison between three conditions is done with Mann–Whitney–U‐Test to show the comparison for all four types of studied tumoroids. d) An example showing the dynamics of tumoroid secretion including the raw secretion signal (grey curve) along with smoothing (green curve), and its corresponding real‐time secretion rate (light blue curve) which is obtained by taking the first derivative of the smoothed curve. e) Scatterplots of the standard deviation of secretion rate versus the mean secretion rate for all four tumoroid types along with the histogram shown on the side of each axis.

The automated scanning capabilities of our platform allow to simultaneously measure the secretory behavior of tens of tumoroids at a time. Our experiments focus on the analysis of HCT116 tumoroids and the three PDTs under various conditions, namely normoxia, hypoxia (1% oxygen), and hypoxia with ActD pre‐treatment. **Figure** **5**a displays the individual temporal sensorgram curves of all conditions obtained from each tumoroid sample. Additionally, Figure 5b exhibits a line plot depicting the averaged curves along with the 95% confidence level for different conditions. We observe that in hypoxic conditions, the VEGF‐A secretion signal in HCT116 tumoroids and all PDTs, with the exception of NW, is higher compared to the normoxia condition. Furthermore, under hypoxia, the secretion signal shows reduction when the samples are pre‐treated with 1 µg mL^−1^ ActD for one hour, as indicated by the temporal sensorgrams of MS and NS PDTs. Notably, the absence of reduced VEGF‐A secretion in the NW PDT treated with ActD suggests that this particular patient sample is unlikely to be responsive to this specific drug, in contrast to the other patient samples.

In the case of HCT116 tumoroids, we observe that the secretion of VEGF‐A initially appears to be suppressed within the first 10 h, as indicated by the considerably lower values in the sensorgrams (Figure 5b, top panel). However, after this initial period, the secretion rate of VEGF‐A starts to rise significantly, ultimately surpassing both the hypoxia and normoxia conditions after 15 h of ActD treatment. This observation could be related to the morphology of the HCT116 cell line derived tumoroids featuring a dense structure without lumens (Figure S9, Supporting Information). This structure could lead to the exposure of the drug mostly to the cells located in the outer region while preserving the capability of inner cells to secrete VEGF.

To further analyze the effects of different treatments on VEGF‐A secretion, we calculate the secretion levels for each tumoroid at the 12 and 20 h time points by averaging the data within the corresponding time ranges (Figure 5c). Notably, the results indicate variations in VEGF‐A secretion between hypoxic and normoxic conditions, as well as between hypoxic conditions with and without ActD pre‐treatment for HCT116, MS PDT, and NS PDT. In contrast, the secretion signals of NW PDT did not exhibit significant variations between the conditions at either 12 or 20 h after the measurement started.

One crucial attribute for comprehending the kinetics of protein secretion is the secretion rate, as it provides insights into the efficiency and speed of protein production and release. Utilizing the real‐time secretion signal at our disposal, an examination of the secretion rate can be conducted to investigate its dynamic variations over time. Figure 5d presents a representative example illustrating the kinetic analysis from the real‐time secretion signal. The raw data (grey curve) is first subjected to smoothing (green curve) and then the first derivative of the smoothed curve is computed to extract the real‐time variation rate of the secretion signal (light blue curve). In this example, the secretion starts to appear after ≈4 h. This signal onset is clearly discernible in the secretion rate curve, where a significant increase coincides with the initiation of the secretion signal. Subsequently, the secretion rate decelerates over the subsequent 10 h.

We performed a comparison of different treatment conditions in HCT116 tumoroids and the three PDTs by creating a scatter plot that depicts the mean value and standard deviation of the secretion rate, and placing the corresponding histograms on the side of each axis (Figure 5e). In these plots, all three PDTs exhibit a distribution of mean and standard deviation closer to the origin when pre‐treated with ActD, in contrast to the hypoxia and normoxia conditions. However, the trend is the opposite in the plot of HCT116 tumoroids, where the ActD pre‐treated tumoroids display a higher mean average rate and a bigger rate variation than the other conditions. Comparing tumoroids under hypoxic and normoxic conditions, we note that hypoxia generally leads to a slightly higher secretion rate, as well as increased rate variation, in almost all patients, with the exception of the NW PDT, where the distribution between hypoxia and normoxia is nearly indistinguishable.

### 2.6 Profiling Dynamic Morphology Changes and Motion of Single CRC Tumoroids

**Figure 6:**
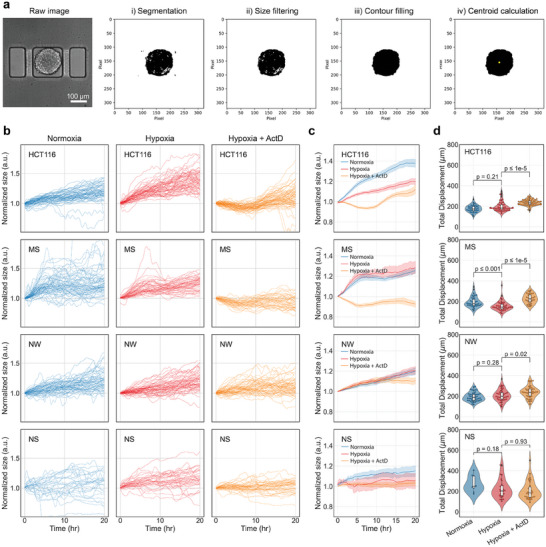
Tumoroid size and motility analysis through machine learning‐assisted segmentation process. a) segmentation image processing of an example tumoroid. The raw image undergoes the steps of i) segmentation, ii) size filtering, iii) contour filling, and iv) centroid calculation. b) Size variation of HCT116 tumoroids and all the PDTs (shown in different rows) over time under various treatment conditions (shown in different columns). c) An averaged curve along with the 95% confidence level plotted in the same graph for four different types of tumoroids. d) Total displacement of each type of tumoroids under various conditions is calculated by summing the displacements between each time step over the 20 h of the measurement.

The dual‐imaging capability of our platform with both spectroscopic and bright‐field imaging enables us to concurrently monitor tumoroid activity in real‐time by analyzing its size and movement together with secretion measurements. Cell motility plays a significant role in both normal physiological functions and pathological conditions, and its investigation has garnered attention to shed light on disease modeling and cancer progression.^[^ ^47^, ^48^, ^49^ ^]^ Likewise, the ability to track the tumor size grown can provide crucial information on the efficacy of cancer drug candidates. In this regard, our dual‐imaging platform offers unique advantages to studying these aspects. To provide a clearer depiction of how the tumoroid morphology evolves over time, the captured images are cropped to create time‐lapse videos, each focusing on a specific microwell containing a tumoroid (Videos S1–S12, Supporting Information). With the total number of patients, tumoroid samples, and time frames in the videos, the system produces an extensive volume of the datasets, which is time‐consuming and prone to inaccuracies to analyze manually. To accurately handle a large number of images and enable precise recognition of tumoroid morphology for a detailed tumor size and movement analysis, machine learning‐assisted approaches for cell recognition, segmentation, and viability analysis can be implemented as reported in related works.^[^ ^50^, ^51^, ^52^ ^]^ Here, we employed machine learning‐assisted image processing through ilastik,^[^ ^53^ ^]^ an open‐source machine learning toolkit designed for image classification and segmentation. With ≈200 cropped frames including HCT116 tumoroids and the PDTs labeled for the training, the pixels are classified into “tumoroid” or “background” highly accurately (Figure S10, Supporting Information). The resulting segmentation images are subjected to further analysis to determine the size and centroid of the tumoroid (**Figure** **6**a).

Figure 6b illustrates the temporal variation in tumoroid size for HCT116 tumoroids and all PDTs under different treatment conditions. It is evident from the plots that tumoroids from all patients demonstrate an increasing size trend over time, regardless of whether they are subjected to hypoxic or normoxic conditions. However, ActD treatment leads to a delay in the size increase or even a reduction in tumoroid size. Our findings correlate well with the cancer‐suppressive capabilities of ActD, as demonstrated through real‐time monitoring of CRC tumoroids. Among the PDTs, NW tumoroids exhibit a comparatively limited suppression of size growth compared to other patients. Although the tumoroid size initially increases over time, it gradually decelerates after 12 h of measurement (Figure 6c). This phenomenon suggests that this particular patient exhibits a slower response to ActD compared to other PDTs and HCT116 tumoroids.

To evaluate tumoroid motility, we utilize the processed binary images of tumoroids to calculate the centroid of each one. By tracking the centroid at each time point, we can quantify the displacement relative to the previous time point. Summing these displacements provides a measure of the total tumoroid displacement, offering insight into the degree of tumoroid motility (Figure 6d). Given that tumoroid movement is confined within microwells, it is reasonable to expect that smaller tumoroids have more space for motion, while larger tumoroids experience limited freedom of movement. Therefore, to ensure a more focused analysis of tumoroid motility and mitigate the impact of tumoroid size on displacements, we analyze only the tumoroids within a specific effective diameter range of 150 ± 20 µm. This enables us to examine tumoroid motility within a defined size window. When comparing different treatment conditions across all patients, we observe that the total displacement is relatively higher in the ActD pre‐treatment condition compared to the hypoxia and normoxia conditions for HCT116 tumoroids, MS PDT, and NW PDT. Whereas for the NS PDT, the overall distribution under all three treatment conditions is wider, which in turn limits to make a clear comparison.

## 3 Conclusion

In this work, we present a label‐free and real‐time analysis of single‐tumoroid secretion, growth, and mobility by utilizing a high‐throughput dual‐imaging optical platform. This platform incorporates an ultrasensitive nanoplasmonic biosensor and a novel PDMS microwell array. The unique two‐layered structure design of the microwell prevents signal disturbance from escaping cells or debris, ensuring accurate secretion analysis. In a microarray format, we analyze tens of tumoroids derived from the HCT116 colorectal cancer cell line, as well as three patient‐derived tumoroids, to assess VEGF‐A secretion in real‐time over 20 h with a 10 min temporal sampling for kinetic analysis.

We evaluate secretion levels, secretion rates, and their variations among different treatment conditions, including hypoxia, normoxia, and hypoxia combined with pre‐treatment of Actinomycin D (ActD), an antibiotic drug known for its anticancer activity. Our results demonstrate the capability of the platform to monitor differences in VEGF‐A secretion under these conditions and investigate the efficacy of ActD in inhibiting growth factor secretion beyond mere viability monitoring. Moreover, the dual‐imaging capability allows for real‐time monitoring of tumoroid morphology, providing valuable insights into tumoroid growth and movement. We observed delayed tumoroid growth or size reduction following drug treatments. The machine learning approach we implemented can not only recognize and analyze tumoroid morphology but also potentially analyze secretion signal patterns for high‐throughput and automated analysis applications.^[^ ^54^ ^]^ In addition, further studies can be done on exploring the relationship between tumoroid sizes and protein secretion patterns, as tumoroid secretion and functional behaviors are influenced by their sizes. By offering comprehensive information on both the secretomic and morphological response of tumoroids in different conditions, our biosensing platform exhibits high potential for applications in drug screening and fundamental organoid research.

## 4 Experimental Section

### Nanohole Array Chip Fabrication

The wafer‐level fabrication of gold nanohole array chips was achieved using deep‐UV lithography. Fused silica wafers of 100 mm diameter and 500 µm thickness (University Wafer, Inc.) went through a standard RCA cleaning process before being deposited with 10 nm Ti followed by 120 nm Au using an electron beam evaporator (EVA760, Alliance Concept). The Ti layer was employed to enhance the adhesion of the Au film to the fused silica substrate and suppress irrelevant surface modes induced by the Au/glass interface. After a post clean with RCA‐1 procedure to further remove possible polymer residues, the NHAs were patterned using a 248 nm deep‐UV stepper (PAS 5500/300 DUV, ASML). The NHA pattern on Ti‐Au films was formed by an ion beam etching system (Ionfab 300 Plus, Oxford Instruments plc), followed by a resist stripping process. A second lithography step was done to pattern the identification codes for each chip. This step was done using a mask‐less direct laser writer (MLA150, Heidelberg Instruments) and followed by gold wet etching using an iodine‐based gold etchant (TechniEtch ACI2, Microchemicals GmbH). The identification codes help us to track and characterize the chip performance further and ensure consistent results. The wafer was then diced into 1 cm × 1 cm chip using a dicing saw (DAD321, DISCO Corporation), and the photoresist from the second lithography step was removed with a positive photoresist remover solution (MICROPOSIT Remover 1165, Rohm & Haas Electronic Materials LLC) at 70 °C and 1 h of sonication for twice. After an oxygen plasma cleaning for 2 min (Tepla 300, VA TePla America) and an RCA‐1 clean for 15 min, the chips were stored for future use.

### PDMS Microwell Array Fabrication

The fabrication process of the two‐layer PDMS microwell mesh involved a replica molding technique using a master mold with two layers of structures. First, the interconnecting channel part with pillar arrays was patterned on a 4‐inch silicon substrate using a direct laser writer. The substrate was then subjected to a silicon deep reactive‐ion etching Bosch process (Adixen AMS200), creating a 25 µm deep pillar profile with 4 µm spacing between pillars. Following this, an oxygen plasma treatment was performed for both resist stripping and surface dehydration. A layer of SU8 negative epoxy photoresist with a thickness of 250 µm (SU8 3050, Kayaku Advanced Materials, Inc.) was applied in a double‐coating process that included two consecutive coating and soft bake steps. The SU8 layer was then exposed by a mask aligner (MA6Gen3, SÜSS MicroTec SE) at i‐line (365 nm) to pattern the microwell structures. Then, a post‐exposure bake was done to cross‐link the resist, and a development process was used to reveal the design on the substrate.

Prior to being utilized as a master mold, the substrate underwent an anti‐stiction silanization process by trimethylchlorosilane (Merck & Co., Inc.) evaporation. The mixture of base PDMS (Sylgard 184 silicone, Dow Corning Corp.) and the cross‐linker (Sylgard curing agent, Dow Corning Corp.) with a weight ratio of 10:1 was then poured into the master mold, followed by a degassing step to avoid bubbles getting trapped in the small pillar structures. A safe cutting blade (Slice) was used to scrape excess PDMS, leaving only a thin layer on top of the SU8 structures before being cured in an oven at 80 °C for 2 h. Next, the cured PDMS was etched using an ICP‐based high‐density plasma etching system (Advanced Plasma System (APS), SPTS Technologies Ltd.) to reveal the SU8 structures, resulting in the microwells becoming open‐topped. Finally, the peeled PDMS membrane consisting of microwells connected by channels with micropillar arrays was attached to the functionalized AuNHA surface for tumoroid secretion analysis.

### CRC Cell Culture and Tumoroid Generation

HCT116 (human colorectal carcinoma) cell line (ATCC) was cultured in 25 cm^2^ culture flasks (Nunc, Thermo Fisher Scientific Inc.) using Dulbecco's Modified Eagle Media (DMEM, Gibco) with an additional supplement of 10% FBS (Gibco), 1% penicillin/streptomycin (Gibco), and 10 mm HEPES buffer (Gibco). Cells were incubated at 37 °C in an incubator with 5% CO2, and were subcultured every 2–3 days using TrypLE Express (Gibco) and diluted with growth medium at a ratio of 1:5 to 1:10. For the tumoroid generation, cells were collected from the culture at the confluency of >80%.

HCT116 tumoroids were formed in the 96‐well plate with hydrogel microwell arrays on the bottom (Grid3D, SUN bioscience SA). Trypsinized cells from the culture were collected and centrifuged, then resuspended in the tumoroid expansion medium comprising of Advanced DMEM/F12 (Gibco), Glutamax (Gibco), 10 mm HEPES buffer (Gibco), 1% penicillin/streptomycin (Gibco), B‐27 (Gibco), Primocin (InvivoGen), and 2 µm Thiazovivin (Sigma–Aldrich) at an appropriate density to deposit ≈300 cells per microwell. 50 µL of the cell suspension was added onto each microwell array and waited for 20 min for cells to settle to the bottom. 150 µL of expansion medium was then added gently to the array, and the plate was incubated at 37 °C in an incubator with 5% CO2. One‐third of the medium was replaced every 2–3 days, and the tumoroids were collected for the on‐chip measurement when the majority of the diameters reached 130–150 µm (3–4 days after seeding).

Patient samples were collected by the Centre Hospitalier Universitaire de Lausanne (CHUV) with informed consent under the protocol CHUV_DO_CTE_TRP_0001_2017 – Tumor MicroEnvironment characterization and ex vivo immune intervention. Patient‐derived human CRC tumoroids were grown and expanded in growth factor‐reduced Matrigel (Corning, 356231) drops of 20 µL in the well of a 24‐well culture plate (Falcon, Corning). The Matrigel drops were immersed in the tumoroid expansion medium consisting of Advanced DMEM/F12 (Gibco), Glutamax (Gibco), 10 mm HEPES buffer (Gibco), 1% penicillin/streptomycin (Gibco), B‐27 (Gibco), Primocin (InvivoGen), and 2 µm Thiazovivin. The full medium was replaced every 2–3 days, and the cells were passaged every 5–7 days by breaking the Matrigel, transferring to a new Falcon tube, and centrifuging at 1500 rpm for 3 min at 4 °C. The tumoroids were dissociated with TrypLE Express (Gibco) at 37 °C into small fragments, and trypsinization was stopped by adding DMEM (Gibco) with 10% FBS (Gibco). The suspension was centrifuged at 1500 rpm for 3 min at 4 °C, and the supernatant was removed completely with care before adding Matrigel. The Matrigel mixed well with the fragments was pipetted as 20 µL drops back in the well plate. After incubating for 15 min at 37 °C for the gelation to occur, the tumoroid expansion medium was added to immerse the drops.

Uniform‐sized human CRC tumoroids were generated using the aforementioned plate with hydrogel microwell arrays. The Matrigel domes were broken and the tumoroids were dissociated with the method mentioned above, but with an extended time to have single cells. The cells were then centrifuged and resuspended at the appropriate density to deposit ≈300 cells per microwell. After 20 min of cell sedimentation in the incubator, 150 µL of expansion medium with 2% Matrigel was added gently to the array. The plate was incubated at 37 °C in an incubator with 5% CO2, and one‐third of the medium was replaced by the expansion medium with 1% Matrigel every 2–3 days. When the majority of the diameters reached 130–150 µm (normally 4–6 days, depending on the patient), the tumoroids were collected for the on‐chip measurement.

### Biosensor Surface Functionalization and Sensor Calibration

The AuNHA biosensor chip was subjected to a cleaning and thiolation procedure, as previously described.^[^ ^34^ ^]^ The thiolation process was carried out for 12–20 h in a nitrogen glove box, using 0.1 mm biotin PEGylated thiol (Biotin‐PEG‐SH) and 0.9 mm hydroxyl PEG thiol (OH‐PEG‐SH) (Prochimia Surfaces). The resulting chip was then rinsed with ethanol, dried with nitrogen gas, and incubated with 100 µg mL^−1^ streptavidin solution (Life Technologies) in phosphate‐buffered saline (PBS) for 2 h at room temperature. Following this step, the chip was washed with PBS three times to remove any unbound streptavidin and dried. The chip was then incubated overnight at 4 °C with a solution of biotinylated VEGF‐A antibody (Abcam plc., ab83132) at a concentration of 50 µg mL^−1^, by adding the solution on top of the chip and allowing it to incubate in a sealed petri dish. After a quick rinse with Milli‐Q water and gentle drying with nitrogen gas, the functionalized chip surface was blocked using 1× clear milk blocking buffer (Thermo Scientific) for 10 min at room temperature. The blocking step was followed by three wash steps with PBS and drying, leaving a chip ready to be assembled with the PDMS microwell for tumoroid seeding and downstream measurements.

The biosensor calibration was conducted by assembling the AuNHA chip with microfluidic channels measuring 500 µm in width and 80 µm in height. Recombinant human VEGF‐A proteins (Abcam plc.) were then injected into the channels using a syringe pump (PHD ULTRA, Harvard Apparatus) at various concentrations diluted with PBS, starting from the lowest concentration to the highest in a cumulative manner. The limit of detection (LOD) of the biosensor was defined as LOD = 3.3σ/S, where σ represents the standard deviation of the response and S is the slope of the calibration curve at the lower concentration range. The chip integration configuration and the resulting calibration curve are depicted in Figure S6 (Supporting Information). 

### Tumoroid Retrieval and Seeding on the Integrated Biochip

The AuNHA chip, attached with a PDMS microwell array, was affixed to the bottom glass of a chamber slide (µ‐Slide 2 Well, ibidi GMBH) using a thin PDMS layer, given the sticky nature of PDMS. Following immersion of the chip in Advanced DMEM/F12 basal medium (Gibco) and degassing to eliminate air bubbles trapped in the PDMS microwells, the chip was ready for tumoroid seeding.

The cultured tumoroids were retrieved from the hydrogel microwell arrays for downstream on‐chip measurements by pipetting to resuspend the tumoroids from the bottom of the microwells. This step was repeated until all tumoroids were transferred into a Falcon tube. A cell strainer with a mesh size of 100 µm was then used to exclude single cells, fragments, and small tumoroids (<100 µm). The remaining tumoroids were resuspended and seeded onto the chip with PDMS microwell array by pipetting and allowing them to settle into the microwells. Gentle shaking was applied to move the tumoroids outside of the microwells to empty microwells, thereby increasing the number of tumoroid‐occupied microwells for measurement. The leftover tumoroids were removed and the basal medium was replaced with the expansion medium with or without further conditioning depending on the experiment conditions. The schematics of the procedures to prevent multiple tumoroids from entering the same microwell are illustrated in Figure S11 (Supporting Information). The slide was then moved to the sensing platform and allowed to stabilize for 1–1.5 h at the constant temperature of 37 °C set in the stage‐top incubator before initiating the optical measurements.

### Sensing Platform Setup and Data Processing

The optical measurement setup that enabled simultaneous spectroscopic and bright‐field imaging was previously described in detail.^[^ ^34^ ^]^ The experimental setup utilized normal incident light from a broadband tungsten‐halogen lamp, which was filtered by a bandpass filter (central wavelength 860 nm, FWHM 120 nm, Salvo Technologies Inc.). The light was then directed onto the nanoplasmonic chip, and the transmitted signal was collected using an objective lens (Nikon Plan Fluor 10×, NA = 0.3). The signal was then split by a dual‐camera image splitter (TwinCam, Cairn Research), which was installed on an inverted microscope (Nikon Ti‐U). One of the two beams was directed to a spectrometer (Shamrock 303i, Andor) and a deep‐cooled CCD camera (iKon‐M, Andor) with 1024 × 1024 pixels and 13 × 13 µm^2^ pixel size, generating 1D spectroscopic images through a slit opening with 500 µm width and a grating of 600 lines mm^−1^. The other beam was directed to a monochrome CMOS camera (DS‐Qi2, Nikon) with 4908 × 3264 pixels and 7.3 × 7.3 µm^2^ pixel size to generate optical images. A customized MATLAB graphic user interface was implemented for the data acquisition, real‐time analysis, and display.

The positions of the stage were defined to correspond with the detection wells before conducting the measurement. The interface extracted the spectra of the detection wells with their connected tumoroid wells having single tumoroids inside, which were then corrected by the signal from the reference wells. The reference wells were connected to empty tumoroid wells and were used to account for the effect of systematic drifts. A region‐of‐interest (ROI) was selected on the spectral images to measure and calculate the resonance peak centroid within a fixed wavelength window of 50% peak‐to‐valley intensity. During the course of the experiment, spectroscopic image and bright‐field image files were sorted into their respective folders to prepare them for further processing. Temporal sensorgrams were calculated and plotted in real time. The saved spectroscopic images were analyzed to construct the spatiotemporal sensorgram, which displays the resonance shift across the slit over time with a spatial resolution of ≈0.6 µm. The image acquisition interval of a single stage position was set to 10 min. The data was subsequently upsampled by a factor of 5 using interpolation for both the time and position axes. The signal was smoothed with Lowess smoothing and median smoothing to mitigate noise prior to plotting.

### Immunofluorescence Imaging and Flow Cytometry

The immunofluorescence staining was done in situ on the hydrogel microwell array after tumoroid formation. Tumoroids were washed with 1× PBS and fixed with 4% paraformaldehyde for 30 min at room temperature. After washing three times with 1× PBS, permeabilization was achieved by treating the samples with 0.2% Triton X‐100 in 1× PBS for 30 min at room temperature. The samples were then blocked with 10% serum in 1× PBS containing 0.02% Triton X‐100 (termed blocking buffer) for at least 4 h or overnight at 4 °C. After blocking, primary antibodies against VEGF‐A (host: rabbit, dilution: 1:50, Thermo Fisher Scientific) were incubated with the samples overnight at 4 °C in the blocking buffer. The samples were then washed thoroughly with 1× PBS by replacing the buffer five times, each time waiting for 30 min, before being incubated with secondary antibodies conjugated with DyLight‐488 (Goat anti‐Rabbit IgG, dilution: 1:100) in blocking buffer overnight at 4 °C. After sample washing for 4 h with 1× PBS, Hoechst 33342 (Thermo Fisher Scientific) diluted 2000 times in PBS was added. Finally, tumoroid samples were washed with 1× PBS for 4 h and imaged using a Leica SP8 inverted confocal microscope with a 25× water‐immersion objective (HC FLUOTAR 25×, NA = 0.95). The same protocol of intracellular VEGF‐A staining was used for the sample preparation for flow cytometry analysis.

### Organoid Size Analysis

Tumoroid images were captured using a monochrome sCMOS camera (DS‐Qi2, Nikon) with a 4908 × 3264 pixel resolution and 7.3 × 7.3 µm^2^ pixel size, using a 10× objective lens (Nikon Plan Fluor 10×, NA = 0.3) covering a 2 × 2 well area. These images were cropped to isolate microwells containing tumoroids and processed with ilastik,^[^ ^42^ ^]^ an open‐source image classification and segmentation toolkit.

To train the classifier, 212 cropped images from different time instances of single microwells containing HCT116 or patient‐derived tumoroids were randomly selected. Pixel classification utilized six features per pixel, including Gaussian, Laplacian of Gaussian, Gaussian gradient magnitude, difference of Gaussians, structure tensor eigenvalues, and Hessian of Gaussian eigenvalues. Gaussian features were computed with seven kernel radii (0.3, 0.7, 1.0, 1.6, 3.5, 5.0, and 10.0 pixels), while the other features used six kernel radii (0.7, 1.0, 1.6, 3.5, 5.0, and 10.0 pixels), resulting in a 37‐value vector for each pixel. During training, human annotators labeled pixels as “tumoroid” or “background.” A random forest classifier with 100 trees was then used to classify the remaining pixels, producing binary “tumoroid”‐“background” masks for each frame.

Subsequently, Python was employed to analyze the binary images, determining tumoroid size and centroid. Connected components were identified using the OpenCV library, and all components except the largest one were removed. The largest contour was filled to correct background pixels within the contour, resulting in a single filled area representing the tumoroid. The tumoroid area was calculated by summing the pixels within this filled contour and then converted from pixel^2^ to µm^2^ using the known pixel size. Centroid coordinates were determined frame by frame to calculate their displacements.

## Conflict of Interest

The authors declare no conflict of interest.

## Supporting information

Supporting Information

Supplemental Video 1

Supplemental Video 2

Supplemental Video 3

Supplemental Video 4

Supplemental Video 5

Supplemental Video 6

Supplemental Video 7

Supplemental Video 8

Supplemental Video 9

Supplemental Video 10

Supplemental Video 11

Supplemental Video 12

Supplemental Video 13

## Data Availability

The data that support the findings of this study are available from the corresponding author upon reasonable request.
