# Supplementary material for: Nanoplasmonic Single‐Tumoroid Microarray for Real‐Time Secretion Analysis
Source: Adv Sci (Weinh). 2024 Jun 24;11(34):2401539. doi: 10.1002/advs.202401539 (PMC11425908; doi:10.1002/advs.202401539)
Supplement: Supplementary file 1 — Supporting Information [file ADVS-11-2401539-s009.docx]

Supporting Information

Nanoplasmonic single-tumoroid microarray for real-time secretion analysis

Yen-Cheng Liu, Saeid Ansaryan, Jiayi Tan, Nicolas Broguiere, Luis Francisco Lorenzo-Martín, Krisztian Homicsko, George Coukos, Matthias P. Lütolf, and Hatice Altug*

**This PDF file includes:**

Figures. S1 to S11

Movies S1 to S13


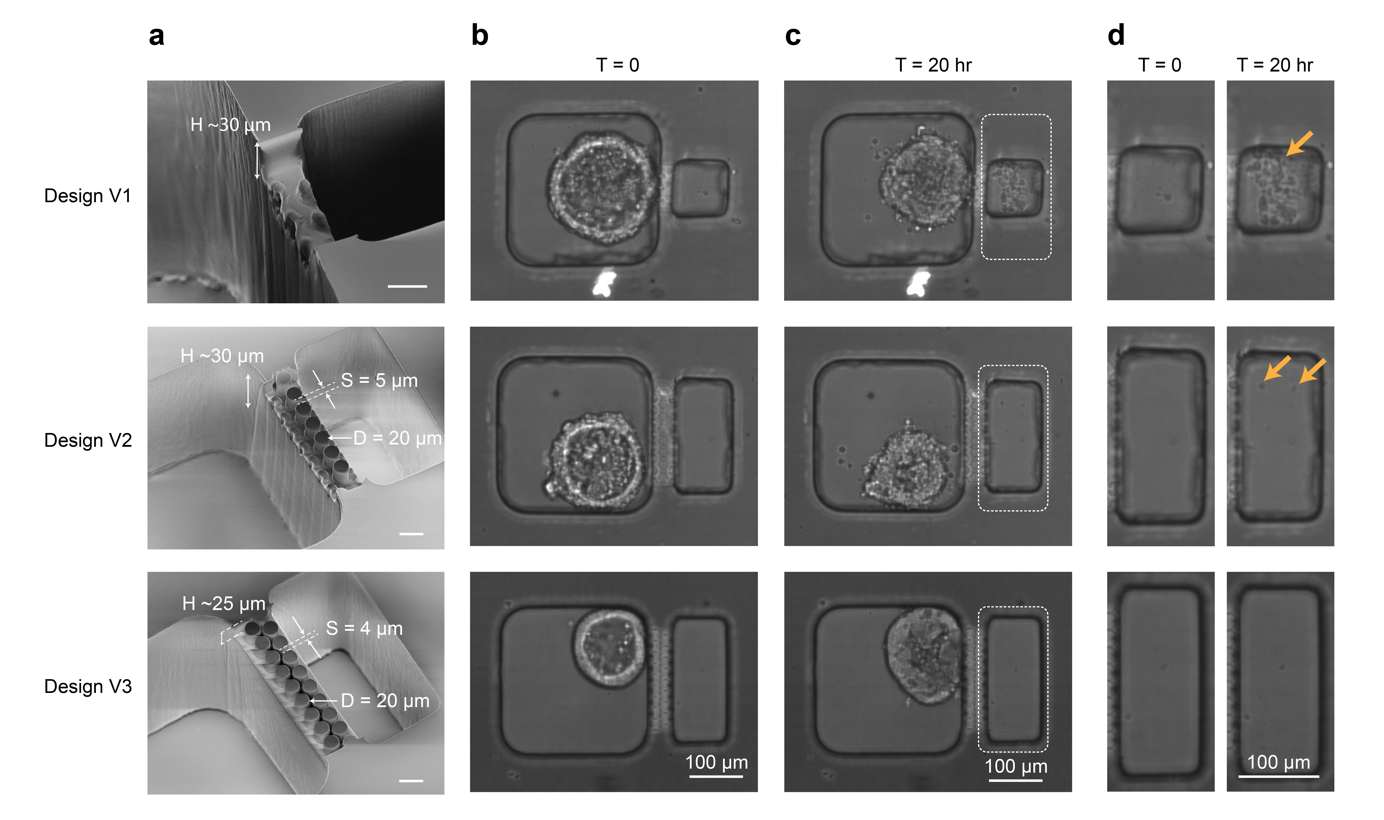


**Figure S1. Design improvement of micropillars for detaching cell blocking.** (a) SEM images of micropillars in each version with design parameters annotated. Scale bar = 30 μm. (b) Optical images of the microwell unit with a tumoroid in the tumoroid well at T=0 for each version. (c) The same microwell images for each version at T=20 hr, with white dashed lines highlighting the detection well for the comparison of debris level inside. d) Zoomed-in images of the detection well area in the end of the measurement. Compared to the design V3, the designs V1 and V2 show debris entering the detection wells after long-term measurement.


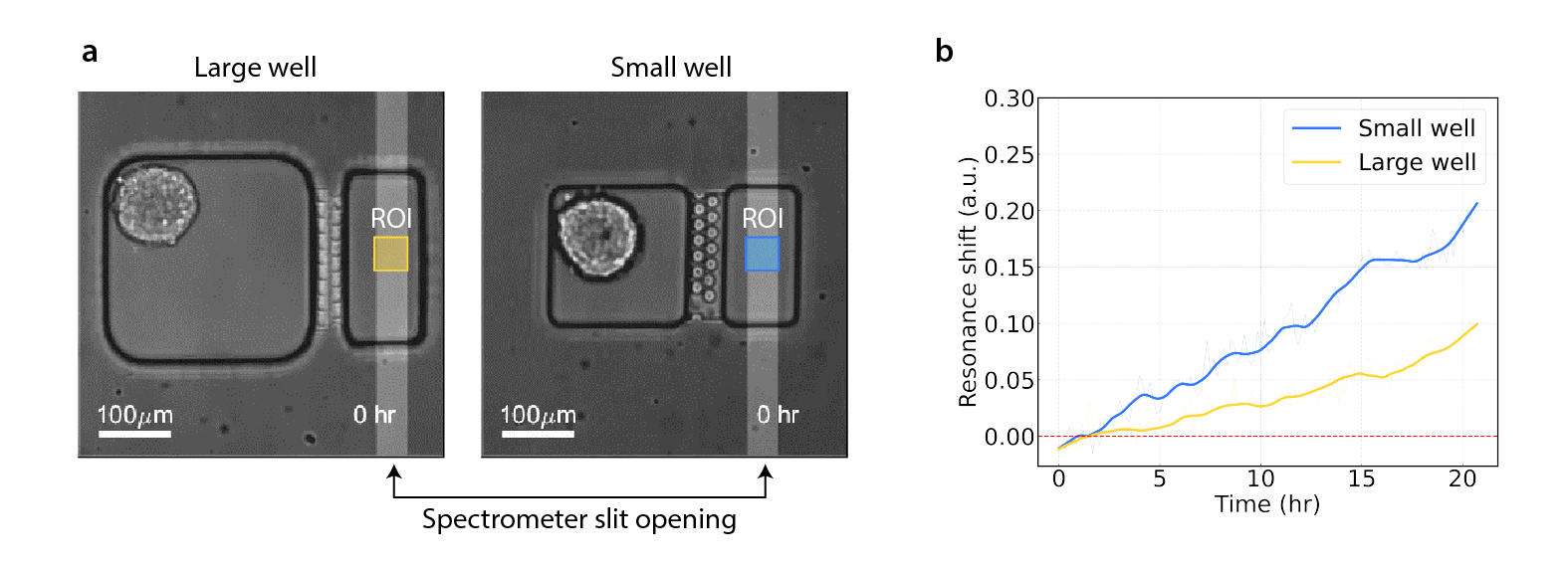


**Figure S2. Effect of tumoroid well size on the secretion signal level.** (a) Microscope images of tumoroids in two different sizes of the tumoroid wells at T=0 with the spectrometer slit opening overlaying with the detection wells and ROIs annotated. (b) VEGF-A secretion signal within the ROIs with the corresponding color over time. The tumoroid in a smaller well shows a higher secretion signal likely due to its proximity to the detection well.


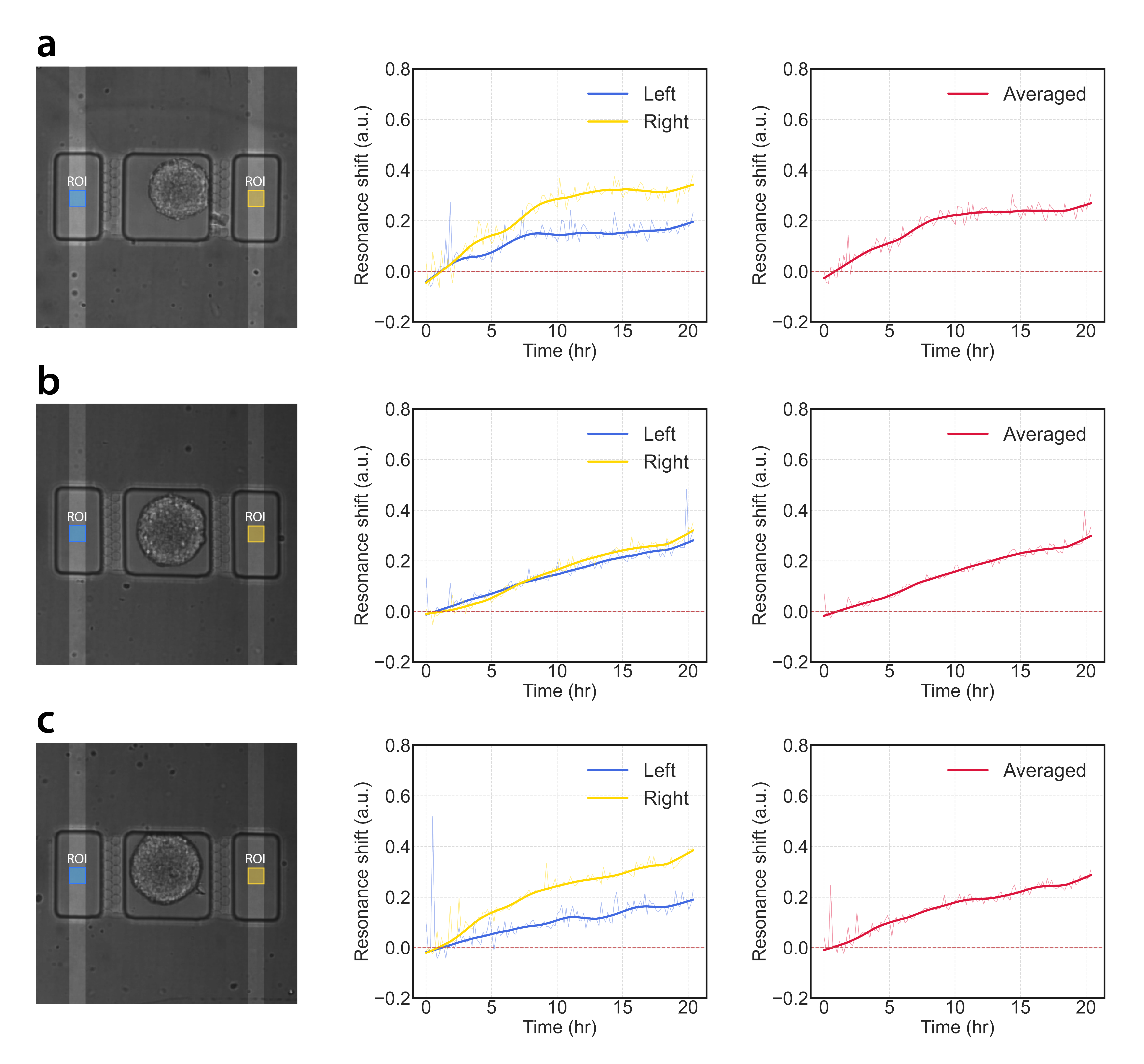


**Figure S3.** **Secretion signal variation in the two detection wells.** (a) Smaller tumoroid locating closer to the detection well on the right than the one on the left, resulting in a higher signal in the detection well on the right. Left panel: optical image of the tumoroid in the microwell at T=0, Mid panel: two signal curves from selected ROIs with the line color same as the corresponded ROI colors. Right panel: Averaging the two curves results in the representing signal curve. (b) Larger tumoroid locating in the center of the tumoroid well, resulting in a similar signal curve in the two detection wells. (c) Larger tumoroid locating in the center of the tumoroid well, but showing different signal curves in the two detection wells.


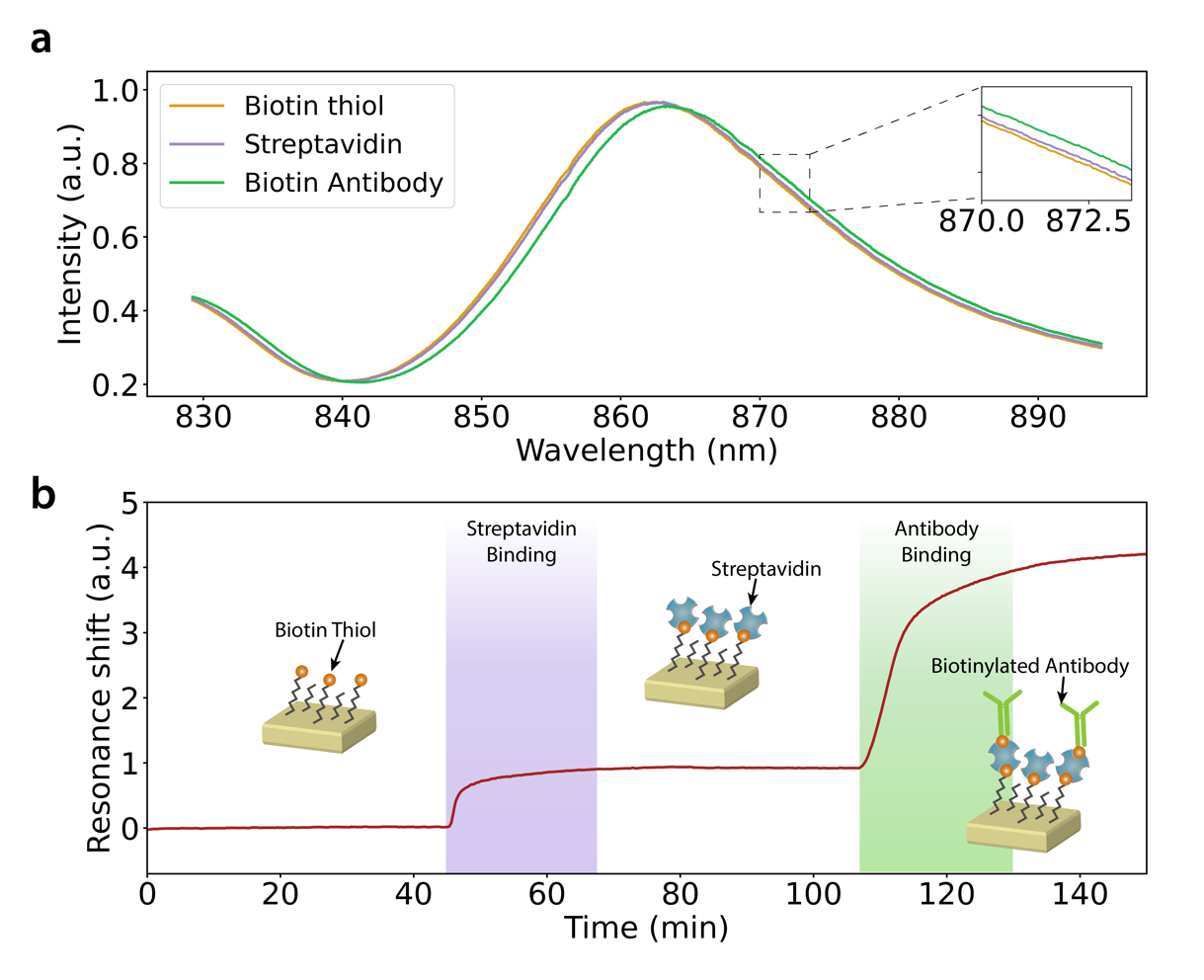


**Figure S4.** **Validation of AuNHA surface functionalization for VEGF-A detection.** (a) EOT spectra of the AuNHA after biotin PEGylated thiol, streptavidin, and biotinylated VEGF-A antibody immobilization. (b) Real-time sensorgram of the EOT spectral shift of thiolated NHA surface reacted with streptavidin and biotinylated antibody.


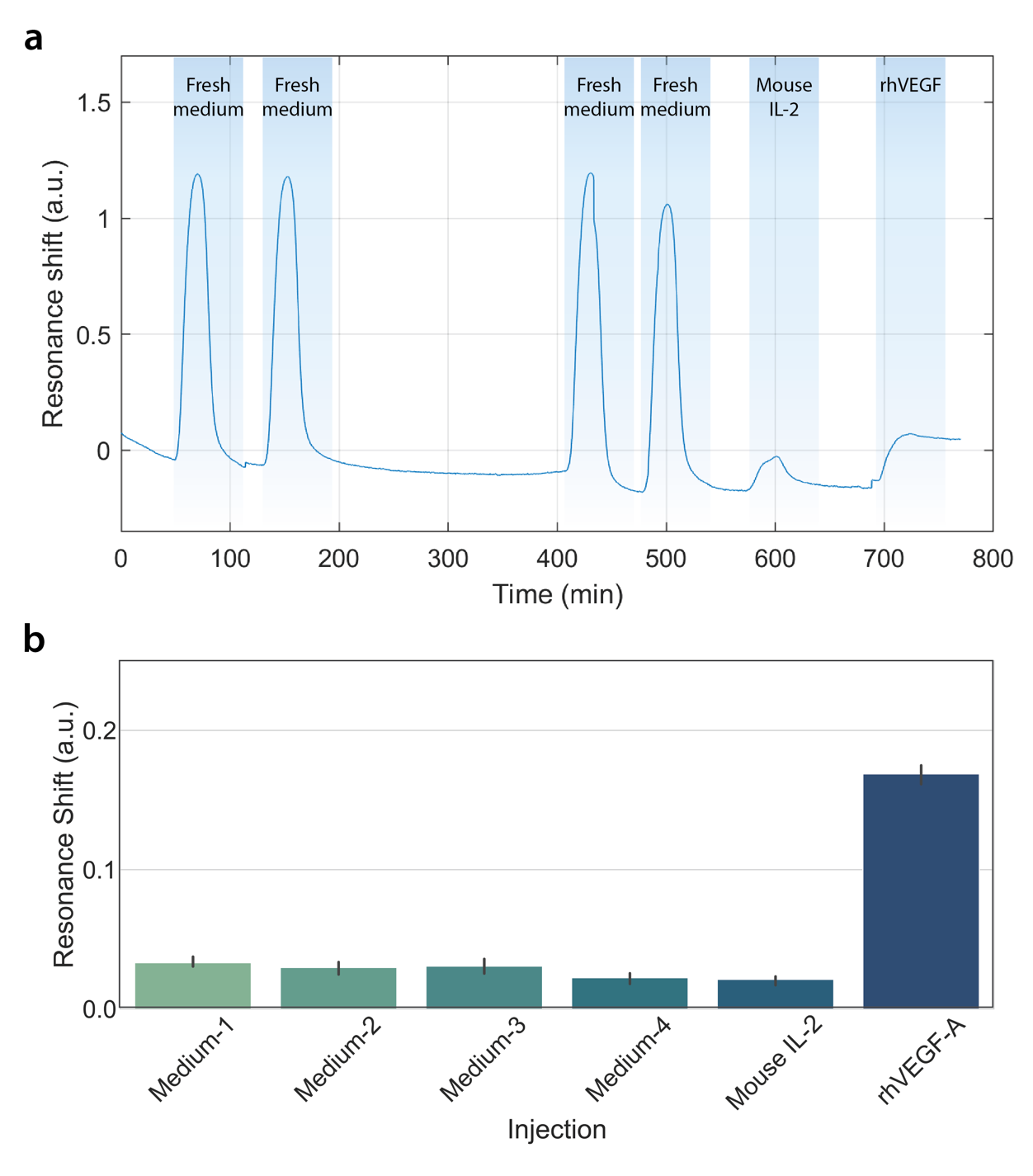


**Figure S5.** **Specificity test of functionalized and blocked AuNHA sensor surface for VEGF-A detection.** (a) Real-time sensorgram of the EOT spectral shift with the injection of fresh tumoroid expansion medium multiple times and mouse interleukin-2 (IL-2) as irrelevant substance, and recombinant human VEGF-A as the analyte. The chip was functionalized with VEGF-A antibody and blocked with the blocking milk before the assembly and injection. (b) Resonance shift signal corresponding to each injection with the shift calculation based on the fixed centroid method.


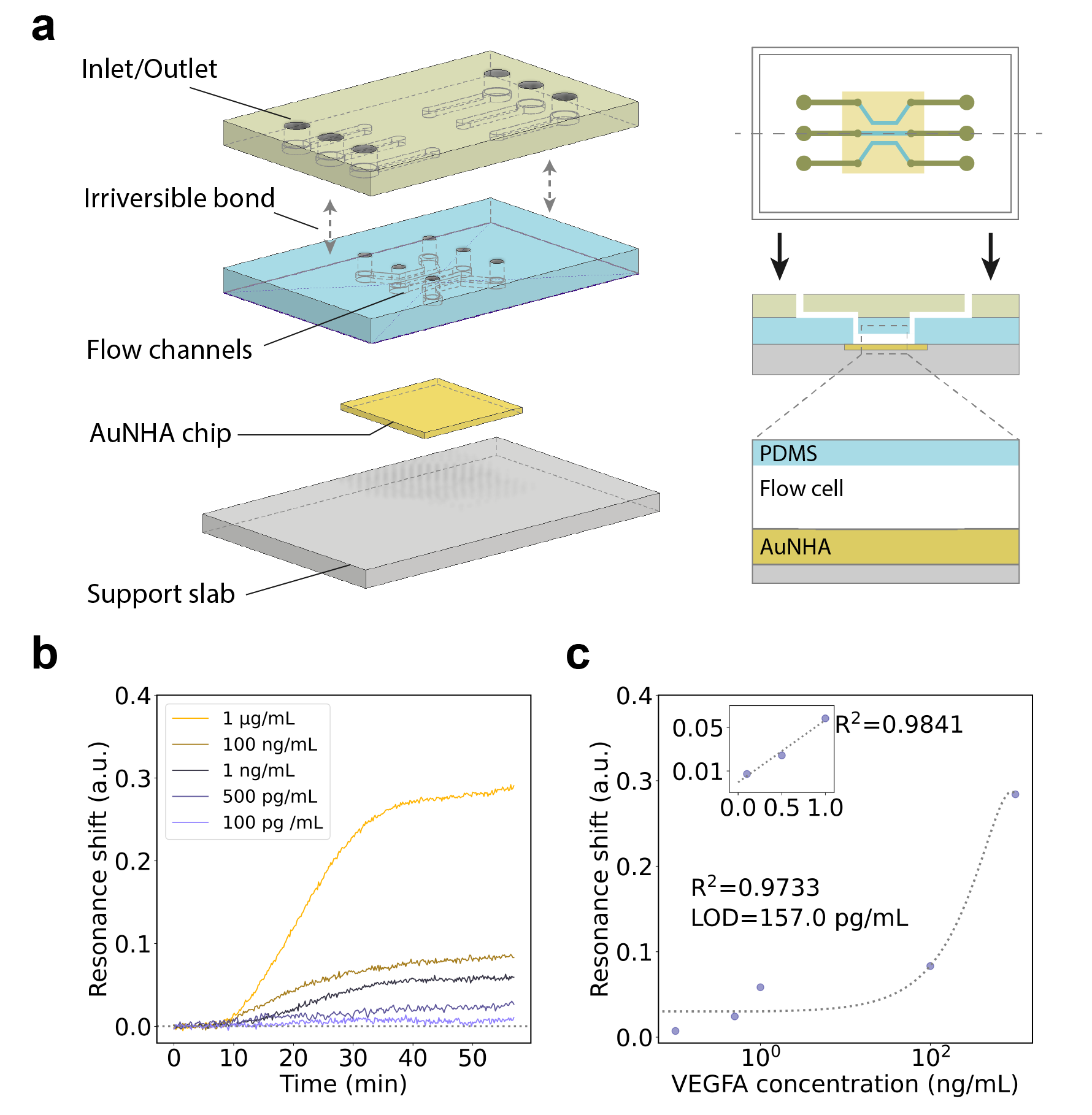


**Figure S6.** **Calibration of the AuNHA biosensor for VEGF-A detection.** a) Assembly of the PDMS microfluidic channels with the nanohole array chip (left panel). The channel design and the cross sections of the assembly are shown in the right panel. b) Real-time sensorgrams of different concentration of VEGF-A ranging from 100 pg/mL to 1 μg/mL. c) Standard calibration curve for label-free detection of VEGF-A molecules. The limit of detection (LOD) is determined as 3.3σ / S, where σ is the standard deviation of the response and S is the slope of the calibration curve at lower concentration range.


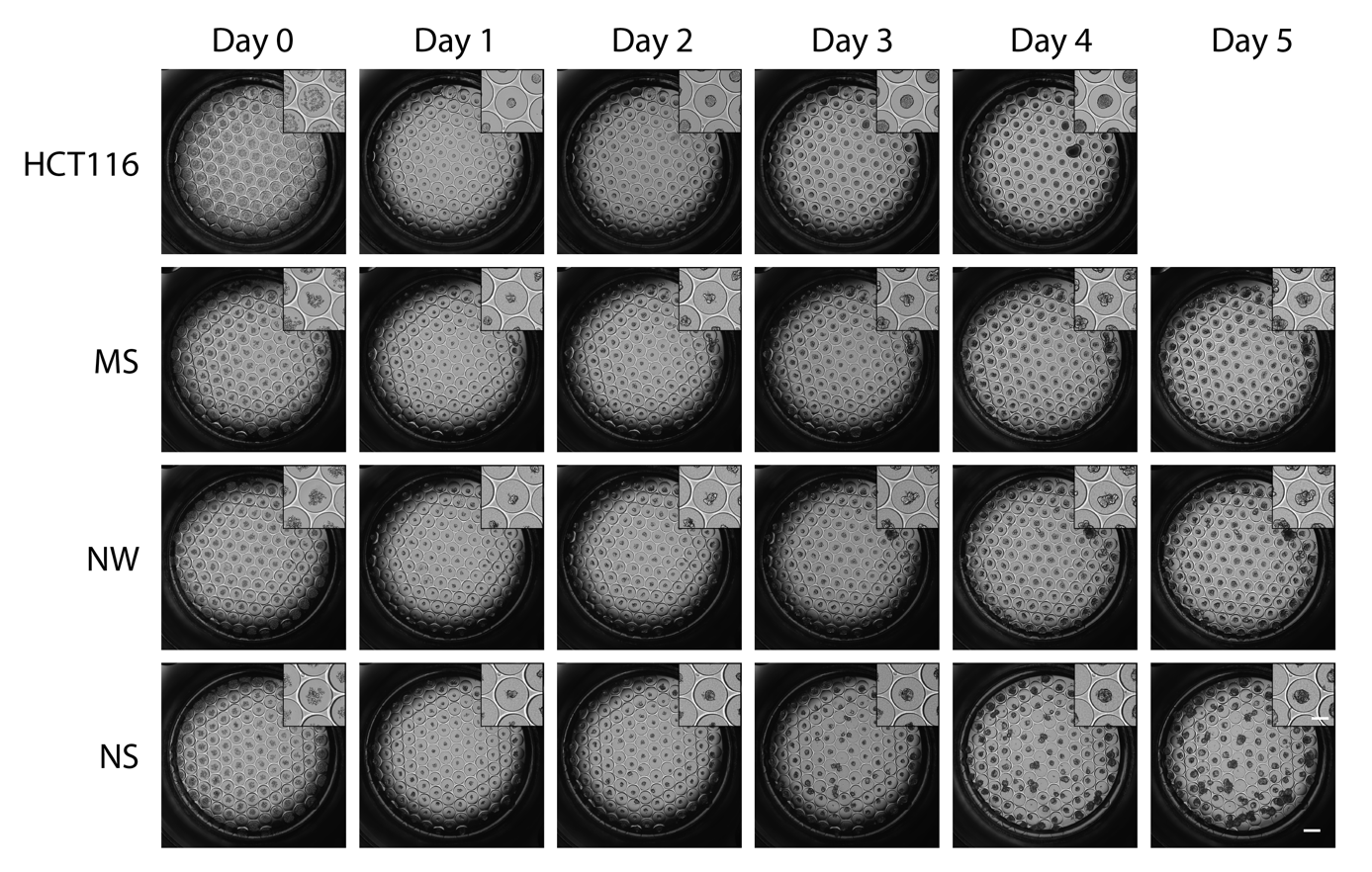


**Figure S7. Tumoroid formation from cell line and different patients in Grid3D plate.** Bright-field microscope images of a single well with 121 hydrogel microwells at various time points following cell seeding. A zoomed-in inset displays a selected microwell. Scale bar: 500 μm (main), 200 μm (inset).


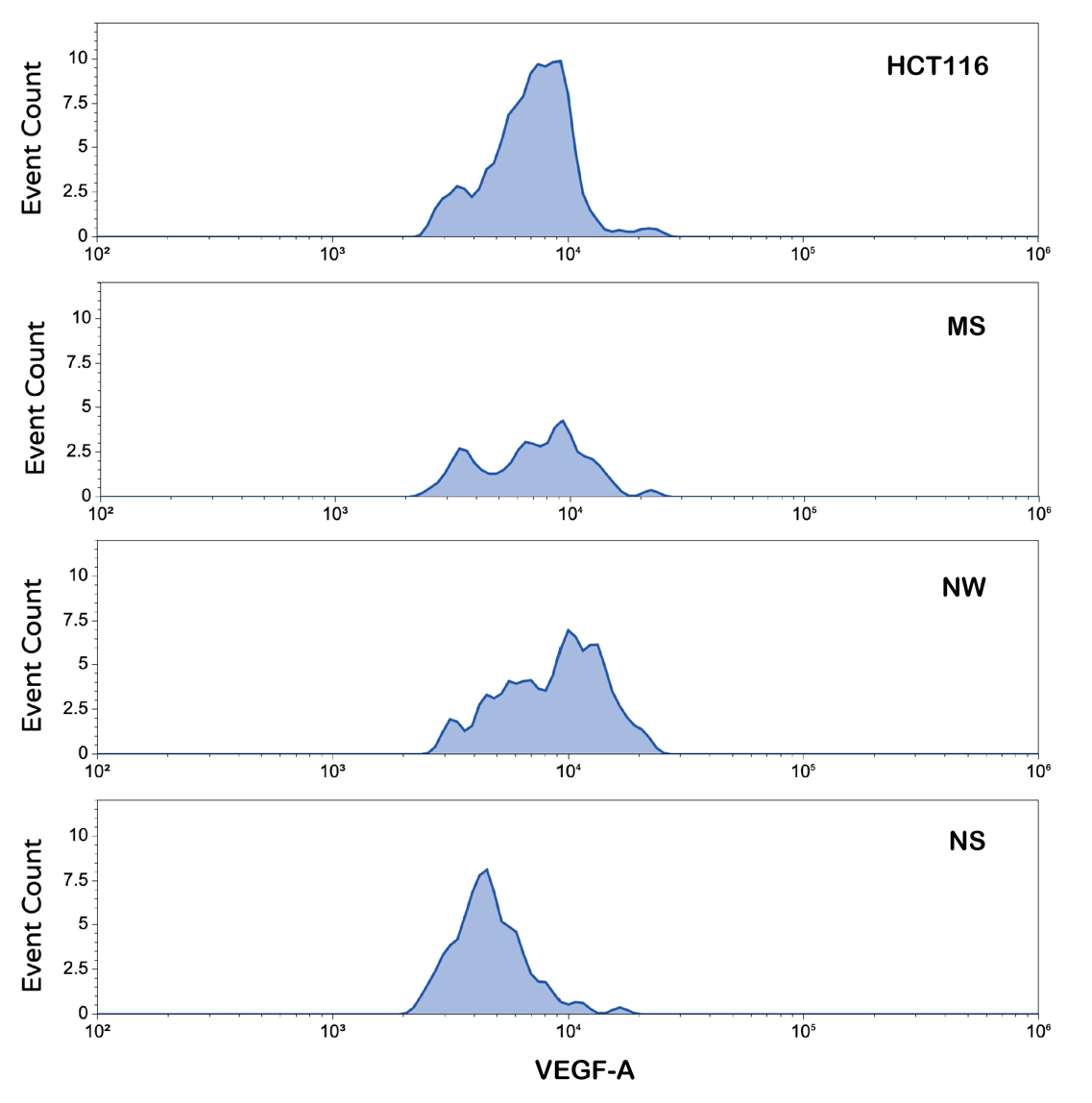


**Figure S8. Large-particle flow cytometry analysis of tumoroids.** Histogram of VEGF-A fluorescence signal from of the tumoroids under normoxic conditions with each tumoroid type in an independent panel.


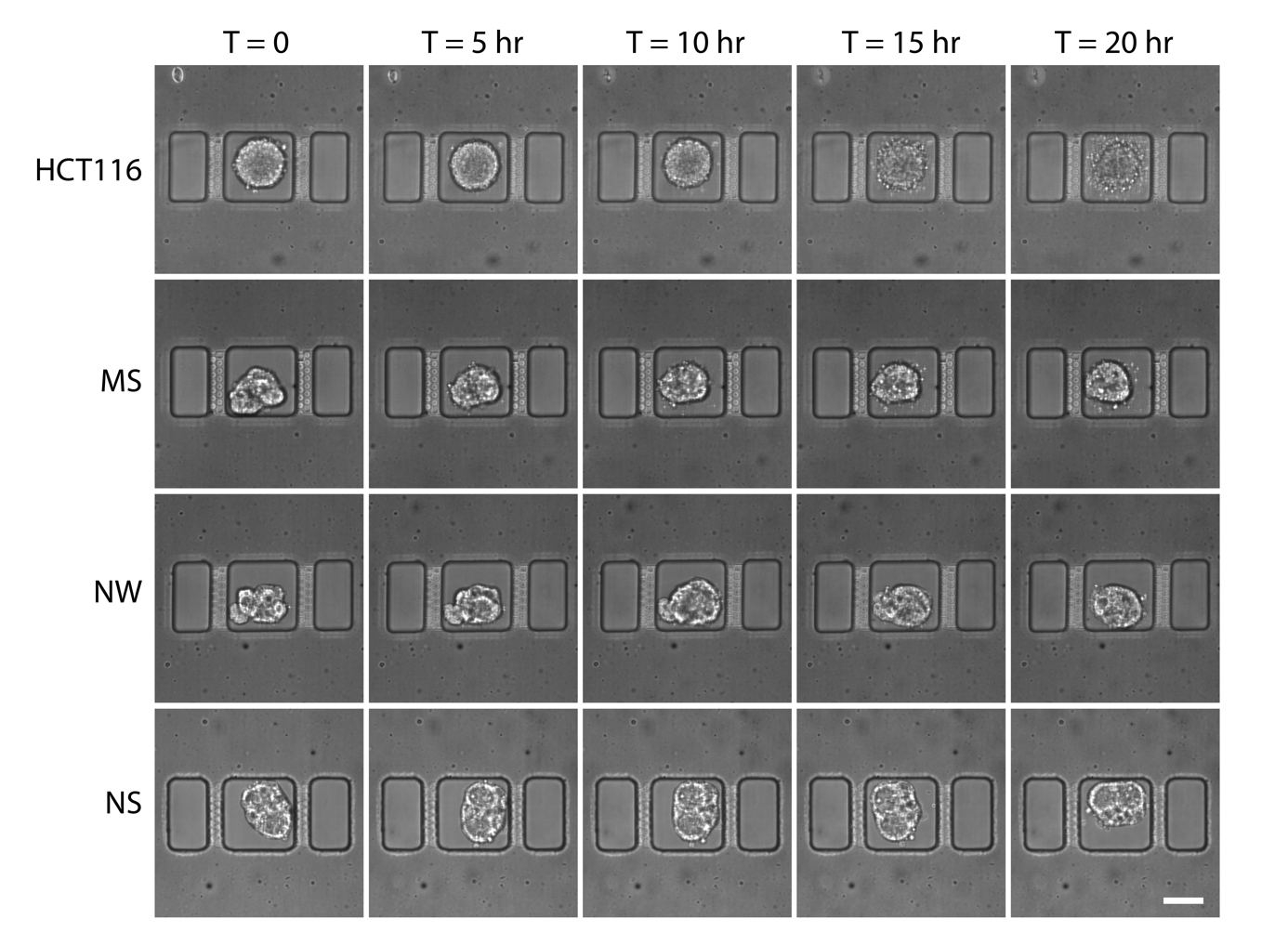


**Figure S9. On-chip tumoroid morphology monitoring after ActD prereatment.** Scale bar = 100 μm.


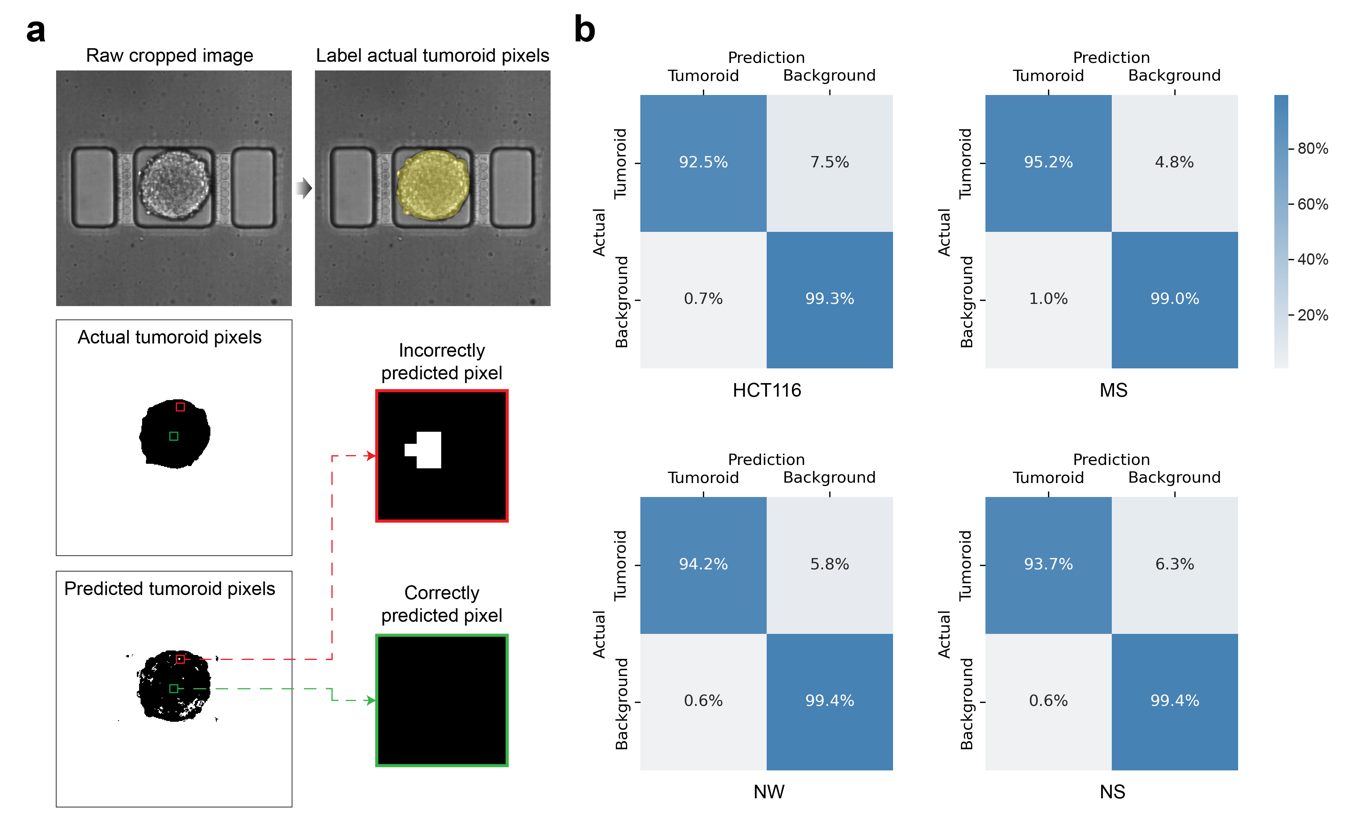


**Figure S10. Prediction accuracy verification of the pixel classification for tumoroid recognition using ilastik.** a) 15 cropped images of 321 × 321 pixel^2^ for each type of tumoroids in the microwells were used for the accuracy verification. The actual tumoroid pixels were labeled manually by human with ilastik, and the exported binary images were used to compare with the analyzed images to calculate the percentage of correctly predicted pixels for both the tumoroid and the background as the accuracy. b) Prediction accuracy tables for each type of tumoroid shows excellent accuracy of at least 92.5% for tumoroids and 99.0% for backgrounds. Number of pixels analyzed: 83574 tumoroid pixels and 1462041 background pixels for HCT116, 96365 tumoroid pixels and 1449250 background pixels for MS, 78562 tumoroid pixels and 1467053 background pixels for NW, and 84435 tumoroid pixels and 1461180 background pixels for NS.


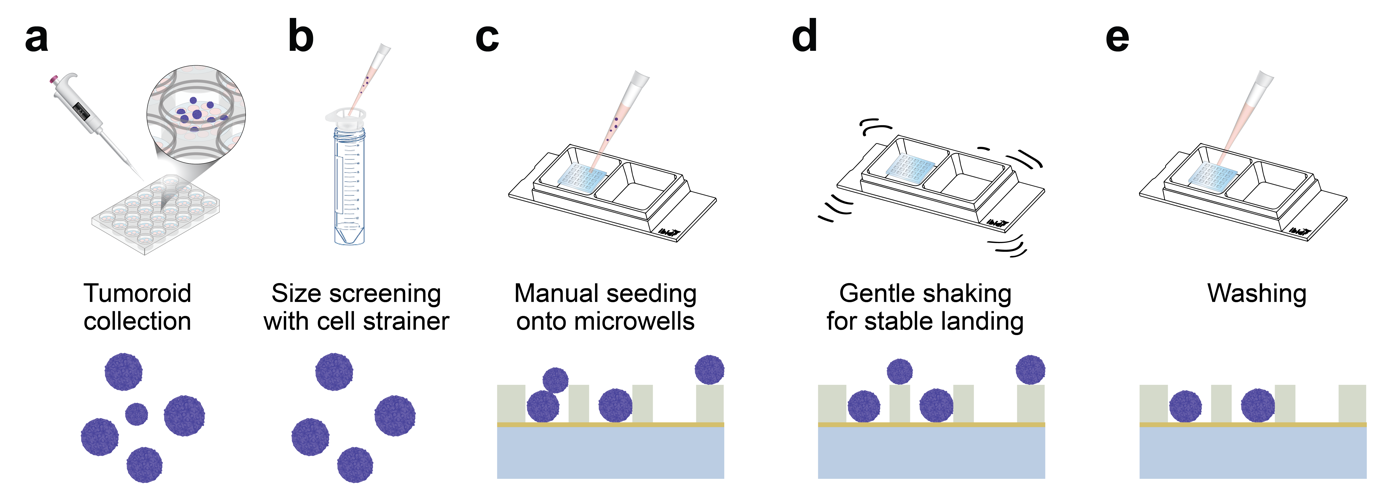


**Figure S11. Tumoroid seeding procedures to prevent multiple tumoroids entering the same microwell.** a) Collection of uniform-sized tumoroids cultured in the Grid3D plate. b) Size screening using a 100 μm-meshed cell strainer to exclude small tumoroids. c) Manual seeding of the tumoroid onto the microwells in the chamber slide using a pipette. d) Gentle shaking to move the second tumoroid stacking on another one out of the microwell. e) Washing with fresh tumoroid expansion medium to remove tumoroids outside of the microwells.

**Movie S1.**

Time-lapse video of the HCT116 tumoroid under normoxia condition.

**Movie S2.**

Time-lapse video of the HCT116 tumoroid under hypoxia condition.

**Movie S3.**

Time-lapse video of the HCT116 tumoroid under hypoxia condition with ActD pre-treatment.

**Movie S4.**

Time-lapse video of the MS PDT under normoxia condition.

**Movie S5.**

Time-lapse video of the MS PDT tumoroid under hypoxia condition.

**Movie S6.**

Time-lapse video of the MS PDT tumoroid under hypoxia condition with ActD pre-treatment.

**Movie S7.**

Time-lapse video of the NW PDT tumoroid under normoxia condition.

**Movie S8.**

Time-lapse video of the NW PDT tumoroid under hypoxia condition.

**Movie S9.**

Time-lapse video of the NW PDT tumoroid under hypoxia condition with ActD pre-treatment.

**Movie S10.**

Time-lapse video of the NS PDT tumoroid under normoxia condition.

**Movie S11.**

Time-lapse video of the NS PDT tumoroid under hypoxia condition.

**Movie S12.**

Time-lapse video of the NS PDT tumoroid under hypoxia condition with ActD pre-treatment.

**Movie S13.**

Screen recording of the real-time tumoroid secretion measurement with automated stage scanning using the MATLAB interface to coordinate the optical and spectroscopic image acquisition.
